# Four new *Microbacterium* species isolated from seaweeds and reclassification of five *Microbacterium* species with a proposal of *Paramicrobacterium* gen. nov. under a genome-based framework of the genus *Microbacterium*

**DOI:** 10.3389/fmicb.2023.1299950

**Published:** 2023-12-18

**Authors:** Soon Dong Lee, Hong Lim Yang, In Seop Kim

**Affiliations:** ^1^Institute of Jeju Microbial Resources, BioPS Co., Ltd., Jeju, Republic of Korea; ^2^Department of Biological Sciences and Biotechnology, Hannam University, Daejon, Republic of Korea; ^3^BioPS Co., Ltd., Daejeon, Republic of Korea

**Keywords:** *Microbacterium*, genome sequencing, core genome analysis, seaweeds, four new species, *Microbacteriaceae*, *Paramicrobacterium* gen. nov., overall phylogenomic clustering

## Abstract

The taxonomic relationships of 10 strains isolated from seaweeds collected from two beaches in Republic of Korea were studied by sequencing and analyses of 16S rRNA genes and whole genomes. For the construction of a more reliable and robust 16S rRNA gene phylogeny, the authentic and nearly complete 16S rRNA gene sequences of all the *Microbacterium* type strains were selected through pairwise comparison of the sequences contained in several public databases including the List of Prokaryotic names with Standing in Nomenclature (LPSN). The clustering of the ten study strains into five distinct groups was apparent in this single gene-based phylogenetic tree. In addition, the 16S rRNA gene sequences of a few type strains were shown to be incorrectly listed in LPSN. An overall phylogenomic clustering of the genus *Microbacterium* was performed with a total of 113 genomes by core genome analysis. As a result, nine major (≥ three type strains) and eight minor (two type strains) clusters were defined mostly at gene support index of 92 and mean intra-cluster OrthoANIu of >80.00%. All of the study strains were assigned to a *Microbacterium liquefaciens* clade and distributed further into four subclusters in the core genome-based phylogenetic tree. *In vitro* phenotypic assays for physiological, biochemical, and chemotaxonomic characteristics were also carried out with the ten study strains and seven closely related type strains. Comparison of the overall genomic relatedness indices (OGRI) including OrthoANIu and digital DNA–DNA hybridization supported that the study strains constituted four new species of the genus *Microbacterium*. In addition, some *Microbacterium* type strains were reclassified as members of preexisting species. Moreover, some of them were embedded in a new genus of the family *Microbacteriaceae* based on their distinct separation in the core genome-based phylogenetic tree and amino acid identity matrices. Based on the results here, four new species, namely, *Microbacterium aurugineum* sp. nov., *Microbacterium croceum* sp. nov., *Microbacterium galbinum* sp. nov., and *Microbacterium sufflavum* sp. nov., are described, along with the proposal of *Paramicrobacterium* gen. nov. containing five reclassified *Microbacterium* species from the “*Microbacterium agarici* clade”, with *Paramicrobacterium agarici* gen. nov., comb. nov. as the type species.

## Introduction

The genus *Microbacterium* Orla-Jensen 1919 (Approved Lists, 1980) emend. Fidalgo et al. (2016) is the type genus of the family *Microbacteriaceae* Park et al., 1995 emend. Zhi et al. (2009) of the order *Microbacteriales* (Salam et al., 2020) and currently contains >130 species with validly published names (https://lpsn.dsmz.de/genus/microbacterium). The genus forms a monophyletic taxon in the phylogenetic trees of the family *Microbacteriaceae* based on the sequences of 16S rRNA genes (Evtushenko, 2012) and housekeeping genes (Richert et al., 2007) but is paraphyletic in whole-genome-based phylogeny (Nouioui et al., 2018). Members of the genus contain different types of peptidoglycan with D-ornithine, L-lysine, or diaminobutyric acid as the diamino acid of the cell wall peptidoglycan (Suzuki and Hamada, 2012; Fidalgo et al., 2016) and can be readily differentiated from other genera of the family *Microbacteriaceae* using a combination of morphological and chemotaxonomic features (Evtushenko, 2012) as well as genome-based phylogeny (Nouioui et al., 2018). The intra-genus phylogenomic relationships have been partly studied, for < 50 species, through the descriptions of new species (Dong et al., 2020; Bellassi et al., 2021; Tian et al., 2021; Xie et al., 2021) or in the whole-genome-based taxonomic classification of the phylum *Actinobacteria* (Nouioui et al., 2018). In the recent descriptions of two new species from larvae of insects (Lee and Kim, 2023), the genomes of 81 *Microbacterium* strains were included in a core genome-based phylogeny.

Members of the genus are widely distributed in diverse environments such as soil, sediment or activated sludge, clinical specimens, plants, freshwater, and seawater (Suzuki and Hamada, 2012). Some species have been isolated from animal feces (Anandham et al., 2011; Dong et al., 2020), plants (Suzuki and Hamada, 2012; Chen et al., 2020), and insects (Steinhaus, 1941; Lysenko, 1959; Heo et al., 2020; Lee and Kim, 2023). About 20% of *Microbacterium* species have been isolated from marine habitats: nine strains from sediments (Suzuki and Hamada, 2012; Yu et al., 2013; Zhang et al., 2014; Mawlankar et al., 2015; Yan et al., 2015; Xie et al., 2021), six from halophyte plants (Alves et al., 2014; Fidalgo et al., 2016; Li et al., 2018; Zhu et al., 2019, 2021), five from seawater (Suzuki and Hamada, 2012; Zhang et al., 2012; Xie et al., 2021), four from marine invertebrates (Kämpfer et al., 2011; Kim et al., 2011; Suzuki and Hamada, 2012; Kaur et al., 2016), and one from marine ascidian (Suzuki and Hamada, 2012). No *Microbacterium* species has been reported to date as isolated from seaweeds.

The aim of this study was to determine the taxonomic relationships of bacterial strains isolated from seaweeds using 16S rRNA gene sequencing and analysis. For an establishment of more reliable and robust 16S rRNA gene phylogeny, the authentic and nearly complete 16S rRNA gene sequences were selected through comparison of the sequences that have been deposited in several public databases for each *Microbacterium* type strain. Whole-genome sequences were obtained for the ten study strains, including *Microbacterium aurantiacum* KACC 20510^T^, *Microbacterium luteolum* KACC 14465^T^, *Microbacterium kitamiense* KACC 20514^T^, and *Microbacterium imperiale* KACC 11896^T^, and analyzed using a combination of the overall genomic relatedness indices (OGRI) and core genome analyses. For these, the 103 genome sequences available for the genus *Microbacterium* (96 type and seven non-type strains) were included in the construction of a core genome-based phylogenomic tree, and the overall phylogenomic clustering of the genus was performed. Moreover, all the *Microbacterium* type strains were compared to evaluate whether they are maintained as separate species using the OGRI including the OrthoANIu, digital DNA–DNA hybridization (dDDH), and amino acid identity (AAI).

## Materials and methods

### Bacterial isolation and maintenance

Ten bacterial strains were isolated from seaweeds collected from Gwakji Beach (33°27′02 N 126°18′14 E) and Samyang Beach (33°31'34“ N 126°35'11” E) in Jeju, Republic of Korea, in 2003 and 2004, respectively. For bacterial isolation, pieces of dried seaweeds were transferred directly onto WAT-SW agar plates (Lee, 2007). Colonies on the plates, incubated at 30°C for 14 days, were subcultured on TSA-SW medium [trypticase soy agar (TSA; Difco) in a mixture of 60% (v/v) natural seawater and 40% (v/v) distilled water]. The pure cultures were maintained in 20% glycerol suspensions supplemented with 60% natural sea water at −20°C and −80°C. The bacterial strains used in this study are listed in Table 1. The reference type strains for phenotypic analyses were obtained from the Korean Agricultural Culture collection (KACC; 11 strains) and German Collection of Microorganisms and Cell Cultures GmbH (DSM; one strain).

**Table 1:** List of bacterial isolates and the *Microbacterium* type strains used in this study, and the accession numbers of their 16S rRNA gene and genome sequences.

**Strain**	**Group^a^**	**Other strain designations^b^**	**16S rRNA gene sequence accession numbers^c^**	**Genome sequence accession numbers^c^**	**Sources and years of isolation**	**Location^d^**
KSW4-10^T^	A	KACC 22272^T^, DSM 112583^T^	MW703964	CP078078	Dried seaweed, 2003	Gwakji Beach, Jeju, ROK
KSW4-16	A	KACC 22273	MW703965	JALPCG010000000	Dried seaweed, 2003	Gwakji Beach, Jeju, ROK
SSW1-7	A	KACC 22274	MW703966	JALPCF010000000	Dried seaweed, 2004	Samyang Beach, Jeju, ROK
SSW1-49^T^	B	KACC 22275^T^, DSM 112581^T^	MW703967	JAHWXN010000000	Dried seaweed, 2004	Samyang Beach, Jeju, ROK
KSW2-24^T^	C	KACC22276^T^, DSM 112584^T^	MW703968	JAHWXM010000000	Dried seaweed, 2003	Gwakji Beach, Jeju, ROK
KSW4-6	C	KACC 22277	MW703969	JAHWXL010000000	Dried seaweed, 2003	Gwakji Beach, Jeju, ROK
SSW1-36	C	KACC 22278	MW703970	CP078077	Dried seaweed, 2004	Samyang Beach, Jeju, ROK
SSW1-47^T^	D	KACC 22279^T^, DSM 112582^T^	MW703971	JAHWXK010000000	Dried seaweed, 2003	Gwakji Beach, Jeju, ROK
SSW1-51	D	KACC 22280	MW703972	CP078076	Dried seaweed, 2004	Samyang Beach, Jeju, ROK
KSW4-4	E	KACC 22430	MZ701896	JAHWXJ010000000	Dried seaweed, 2003	Gwakji Beach, Jeju, ROK
*M. algeriense*		DSM 109018^T^				
*M. aurantiacum*		KACC 20510^T^		JAHWXI010000000		
*M. arthrosphaerae*		KACC 16680^T^	MZ433296			
*M. liquefaciens*		KACC 14464^T^				
*M. luteolum*		KACC 14465^T^	MZ433297	CP078075		
*M. kitamiense*		KACC 20514^T^		JAHWXH010000000		
*M. imperiale*		KACC 11896^T^		JAHWXG010000000		
*M. maritypicum*		KACC 14436^T^				
*M. oxydans*		KACC 14467^T^				
*M. paraoxydans*		KACC 14506^T^				
*M. saperdae*		KACC 14469^T^				
*M. terrae*		KACC 14470^T^	MZ433298			

^a^defined in 16S rRNA gene tree.

^b^KACC, Korean Agricultural Culture collection; DSM, German Collection of Microorganisms and Cell Cultures GmbH.

^c^determined in this study.

^d^ROK, Republic of Korea.

### 16S rRNA gene sequencing and analysis

For sequencing of 16S rRNA genes, the 10 study strains were grown on marine agar at 30°C for 3 days, and *Microbacterium arthrosphaerae* KACC 16680^T^, *M. luteolum* KACC 14465^T^, and *Microbacterium terrae* KACC 14470^T^ were cultivated on trypticase soy agar (Difco) at 30°C for 3 days. Genomic DNA isolation was performed as described previously (Lee et al., 2020). The PCR amplification and sequencing of the 16S rRNA genes were performed using an ABI Prism 3730XL DNA analyzer with a BigDye Terminator kit v.3.1 (Invitrogen, USA) at the SolGent Company Limited (Deajon, Korea). Multiple alignments of the sequences were performed using CLUSTAL_X (Thompson et al., 1997). 16S rRNA gene-based phylogenetic analyses were performed using the neighbor-joining algorithm contained in the PHYLIP (v 3.68) for 1,329 nucleotide positions. Evolutionary distances were calculated using the Jukes–Cantor model (Jukes and Cantor, 1969). Bootstrap analysis (Felsenstein, 1985) was based on 1,000 replicates.

### Quality check of 16S rRNA gene sequences

Many of *Microbacterium* type strains contain one or more 16S rRNA gene sequences in the NCBI database, one of which is also listed in the List of Prokaryotic names with Standing in Nomenclature (LPSN; https://lpsn.dsmz.de/genus/Microbacterium) or EzBioCloud server (http://www.ezbiocloud.net; Yoon et al., 2017a). To find authentic and near full-length sequences, the 16S rRNA gene sequences of each type strain were compared with one another and further with genome-derived sequences, if available. The alignment of the sequences was manually optimized according to the secondary structure of *Escherichia coli* 16S rRNA (Brosius et al., 1978). The 16S rRNA gene sequence similarity was manually calculated after the gaps or nucleotides present only in one sequence were removed using BioEdit ver 7.7.1 or determined using the EzBioCloud server (https://www.ezbiocloud.net/identify).

### Genome sequencing and phylogenomic analysis

The whole genomes of the 10 study strains, together with *M. aurantiacum* KACC 20510^T^, *M. luteolum* KACC 14465^T^, *M. kitamiense* KACC 20514^T^, and *M. imperiale* KACC 11896^T^ (Table 1), were sequenced using the Illumina HiSeq platform. Raw sequence reads were quality-filtered and *de novo* assembled using SPAdes (http://cab.spbu.ru/software/spades/). To check their authenticity, the genome-derived 16S rRNA gene sequences were compared with the corresponding ones determined by Sanger method. The CheckM (Parks et al., 2014) was used in checking the completeness and contamination of the genomes. For phylogenomic analysis, the genome sequences available for the *Microbacterium* type strains were retrieved from the NCBI database. For non-type strains, the only genome sequences that were confirmed for their identity were included in this study. The phylogenomic tree was reconstructed using the Up to date Bacterial Core Gene (UBCG) pipeline (https://www.ezbiocloud.net/tools/ubcg; Na et al., 2018) based on 92 bacterial core genes present as a single copy in all the genomes. The reliability of the tree branches was evaluated using gene support index (GSI).

### OGRI analyses

As one of thresholds for species delineation, the OrthoANIu values were calculated using the ANI Calculator (https://www.ezbiocloud.net/tools/ani; Yoon et al., 2017b). The dDDH values were calculated using the Genome-to-Genome Distance Calculator with built-in settings (GGDC 2.1) (Meier-Kolthoff et al., 2013). The AAI values as a threshold for genus delineation were determined using the Kostas lab AAI calculator web server (http://enve-omics.ce.gatech.edu/aai/; Rodriguez and Konstantinidis, 2014).

### Phenotypic characterization

A set of physiological and biochemical tests were carried out with the 10 study strains and seven closely related type strains (*Microbacterium algeriense* DSM 109018^T^, *Microbacterium liquefaciens* KACC 14464^T^, *M. luteolum* KACC 14465^T^, *Microbacterium maritypicum* KACC 14436^T^, *Microbacterium oxydans* KACC 14467^T^, *Microbacterium paraoxydans* KACC 14506^T^, and *Microbacterium saperdae* KACC 14469^T^) using traditional methods and commercial kits such as API systems (API 20NE, API ZYM, and AMI 50CH) as reported previously (Lee and Kim, 2023), the categories of which included acid production from substrates, enzyme activity, carbon source assimilation, pH and temperature ranges for growth, NaCl tolerance, esculin degradation, glucose fermentation, and indole production. The growth and cultural characteristics for the ten study strains also were checked using marine agar (MA; BD), nutrient agar (NA; BD), R2A agar (BD), and trypticase soy agar (TSA; BD). Cell morphology and motility, catalase and oxidase activities, and Gram staining were examined as described previously (Lee and Kim, 2023). The diamino acid of the cell wall peptidoglycan was determined by reverse-phase HPLC (Waters 2690) of the derivatized amino acid with AccQ-Fluor Reagent (Waters) according to the instructions of the manufacturer, with the purified cell walls (Lee, 2007). The N-glycolylated muramic acid in cell wall peptidoglycan was determined by a colorimetric method (Uchida and Aida, 1977). The preparation of isoprenoid quinones and polar lipids was performed by the integrated procedure of Minnikin et al. (1984). Analysis of isoprenoid quinones was performed using HPLC as described previously (Kroppenstedt, 1985). Analysis of polar lipids was performed by two-dimensional thin-layer chromatography on silica gel G60 plates (Merck) as previously described (Minnikin et al., 1977). For fatty acid analysis, the 10 study strains and seven closely related type strains were grown on trypticase soy agar (Difco) for 1–2 days at 30°C. The cellular fatty acids were extracted, methylated, and analyzed following the instructions of Microbial Identification System (Sherlock v.6.1) with TSBA6 of the MIDI database. The DNA G+C contents were determined using genome sequencing platform.

## Results

### Identification of authentic 16S rRNA gene sequences

Before the 16S rRNA gene analysis, the sequences available for each *Microbacterium* type strain were retrieved from public databases such as the NCBI, LPSN, and EzBioCloud and checked by pairwise comparison. As a result, the 16S rRNA gene sequences of some type strains were found to be incorrectly listed in the LPSN, in contrast to their original descriptions (Yokota et al., 1993a; Takeuchi and Hatano, 1998b; Zlamala et al., 2002; Schippers et al., 2005; Kämpfer et al., 2011; Anand et al., 2012) (Supplementary Table S1).

*Microbacterium aerolatum* (Zlamala et al., 2002) is represented by two 16S rRNA sequences in the NCBI: AJ309929, in its original description and also listed in the LPSN, and MT760116 (*M*. *aerolatum* CCM 4955^T^). The former sequence shares sequence similarity of 99.71% (4 nt differences) with the genome-derived sequence (BJUW01000027) of *M*. *aerolatum* NBRC 103071^T^, while it showed sequence similarity of 98.58% (19 nt differences) with the MT760116 sequence, which is the same as the genome-derived sequence (BJML01000022) of *Microbacterium testaceum* NBRC 12675^T^. On the other hand, the genome-derived 16S rRNA gene sequence (BMCD01000006) of *M*. *aerolatum* CCM 4955^T^ is the same as the genome-derived sequence (BJUW01000027) of *M*. *aerolatum* NBRC 103071^T^. These results indicate that the MT760116 (*M*. *aerolatum* CCM 4955^T^) is not suitable as a reference sequence of *M. aerolatum*.

*Microbacterium amylolyticum* N5^T^ (Anand et al., 2012) is represented by two 16S rRNA sequences in the NCBI: HQ605925 in its original description and MT760185 (*M. amylolyticum* CCM 7881^T^, also listed in the LPSN). The former sequence shares 100% sequence identity with the genome-derived sequence (CP049253) of *M. amylolyticum* DSM 24221^T^, whereas it shows sequence similarity of 94.84% with the latter sequence (MT760185), which is the same as that (FN870023) given in the original description of *M. arthrosphaerae* CC-VM-Y^T^ (Kämpfer et al., 2011). These results show that the MT760185 (*M. amylolyticum* CCM 7881^T^) sequence listed in LPSN should not be used as a reference sequence of *M. amylolyticum*.

The 16S rRNA gene sequence (FN870023) of *M. arthrosphaerae* CC-VM-Y^T^ (Kämpfer et al., 2011), which is very short at the 3′-end, showed low sequence similarity (97.89%; 27 nt differences) with the MT760166 sequence (from *M. arthrosphaerae* CCM 7681^T^) listed in LPSN, sharing 100% identity with that (HE585693) given in the original description of *Microbacterium murale* I-Gi-001^T^ (Kämpfer et al., 2012). The genome sequence of the type strain has not been determined yet. Therefore, we also determined the 16S rRNA gene sequence (MZ433296) of *M. arthrosphaerae* KACC 16680^T^, revealing that it was in agreement with the original one. These results show that the MT760166 (*M. arthrosphaerae* CCM 7681^T^) sequence listed in LPSN should not be used as a reference sequence of *M. arthrosphaerae*.

The original description of *Microbacterium aurum* H-5^T^ did not contain information for its 16S rRNA gene sequence (Yokota et al., 1993a) but is currently represented by the four sequences of the type strain in the NCBI: AB007418 and D21340 (*M. aurum* IFO 15204^T^), Y17229 (*M. aurum* DSM 8600^T^, also listed in LPSN), and MW227637 (*M. aurum* KACC 15219^T^). Among them, the Y17229 shares sequence similarity of 99.72% (4 nt differences) with the genome-derived sequence (CP018762) of *M. aurum* KACC 15219^T^ and was shown as the nearly complete 16S rRNA gene sequence of *M. aurum*. The other two sequences (AB007418 and D21340), which are short and incomplete at the 5′-end, revealed low sequence similarity (95.49% and 98.48%, respectively) with the genome-derived one (CP018762). Moreover, the fourth sequence (MW227637) also indicated low sequence similarity (98.67%; 19 nt differences) with the genome-derived one (CP018762), despite being determined from the same strain (KACC 15219^T^). These results show that the genome-derived 16S rRNA gene sequence is the more suitable reference sequence for *M. aurum*.

The original description of *Microbacterium dextranolyticum* (Yokota et al., 1993a) did not contain a 16S rRNA gene sequence. Currently, three 16S rRNA gene sequences are included in the NCBI: D21341 and AB007417 (*M. dextranolyticum* IFO 14592^T^) and Y17230 (*M. dextranolyticum* DSM 8607^T^, also listed in LPSN). The genome-derived 16S rRNA gene sequence (JAFBBR010000001) of *M. dextranolyticum* DSM 8607^T^ shared sequence similarity of 99.72% (4 nt differences) with the Y17230, while it showed low sequence similarities with the AB007417 (99.51%; 7 nt differences) and D21341 (99.11%; 11 nt differences) sequences. These results show that genome-derived 16S rRNA gene sequence is the more suitable reference sequence for *M. dextranolyticum*.

The 16S rRNA gene sequence (HM222660) of *Microbacterium hydrothermale* 0704CP-2^T^ (Zhang et al., 2014) revealed sequence similarity of 99.39% (9 nt differences) with the genome-derived one (JAODLQ010000012) recently available from the same type strain. This result suggests that the genome-derived 16S rRNA gene sequence is the better reference sequence for *M. hydrothermale*.

*Microbacterium rhizosphaerae* CHO1^T^ (Cho and Lee, 2017) is currently represented by two 16S rRNA sequences in the NCBI: KP722591 from the original description (also listed in LPSN) and LT593972. The former sequence, which is unstable before position 190 (*E. coli* numbering), showed sequence similarity of 98.50% (21 nt differences) with the latter (LT593972). These results show that the LT593972 rather than the KP722591 is the more suitable reference sequence of *M. rhizosphaerae*.

The 16S rRNA gene sequence (MF084212) of *Microbacterium suaedae* YZYP 306^T^ (Zhu et al., 2019) revealed sequence similarity of 99.21% (12 nt differences) with the genome-derived one (PNFD01000009) obtained from the same type strain, suggesting that the latter sequence is the more suitable reference one of *M. suaedae*.

The 16S rRNA gene sequence (X77445) of *M. testaceum* DSM 20166^T^ (Takeuchi and Hatano, 1998b), which is also listed in LPSN, is the same as the genome-derived sequence (BJML01000022) of *M. testaceum* NBRC 12675^T^, while it revealed very low sequence similarity (94.80%) with the MT760091 sequence (*M. testaceum* CCM 2299^T^) which is the same as that (HQ605295) given in the original description of *M. amylolyticum* N5^T^ (Anand et al., 2012). These results indicate that the MT760091 (*M. testaceum* CCM 2299^T^) is not suitable as a reference sequence of *M. testaceum*.

In addition, many type strains of *Microbacterium* species contained slightly inaccurate 16S rRNA gene sequences, which showed differences of one to seven nucleotides depending on the type strains, as compared with genome-derived ones (Supplementary Table S1). These results indicate that for unraveling the more coherent phylogenetic relationship of the genus *Microbacterium* using 16S rRNA gene phylogeny, it can be better to use genome-derived 16S rRNA gene sequences, if available.

### 16S rRNA gene phylogeny

**Figure 1:**
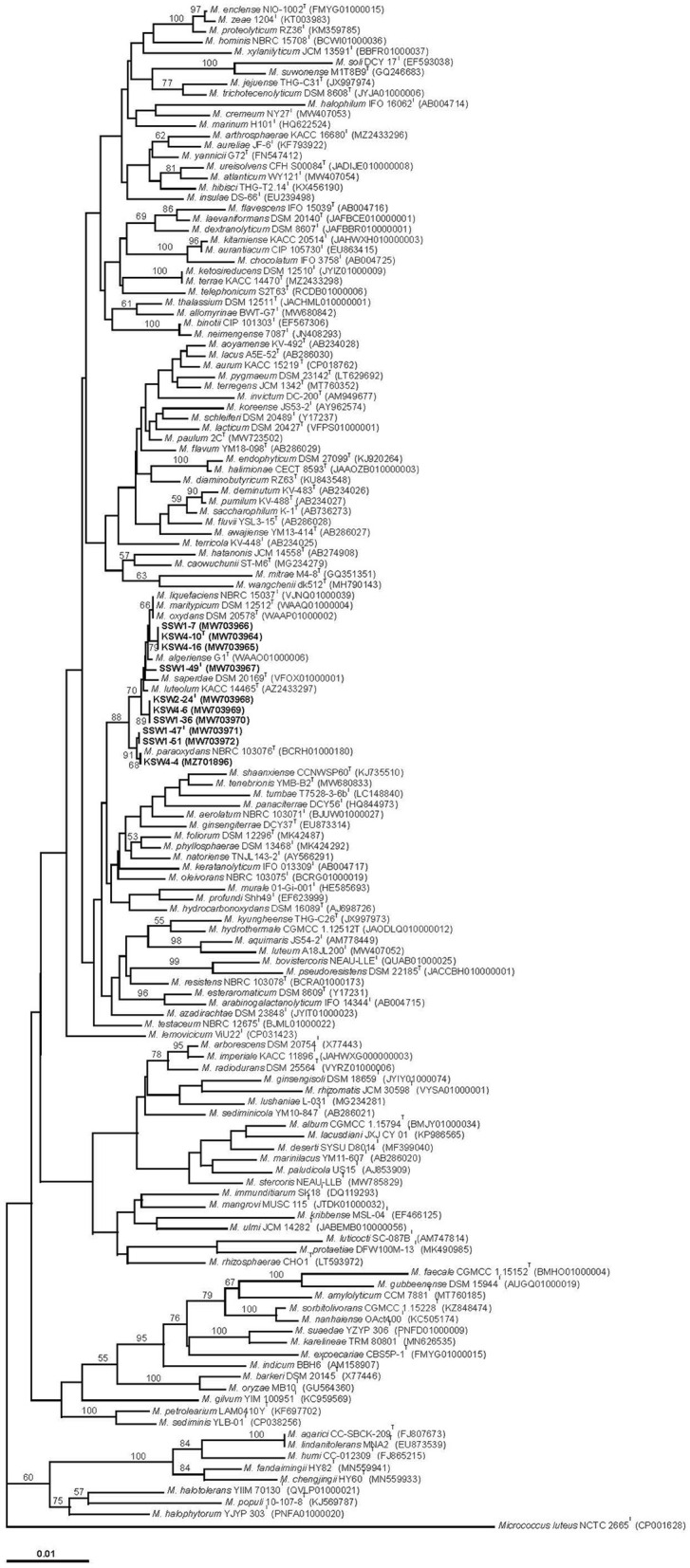
Neighbor-joining phylogenetic tree based on 16S rRNA gene sequences showing the relationships between the 10 study strains and 132 *Microbacterium* type strains. Distances were calculated with the Juke-Cantor model. The tree is based on 1,329 nt. Bootstrap support values >50% (1,000 resamplings) are shown at branches. Bar, 0.01 substitutions per nucleotide position.

A phylogenetic analysis was performed with the 16S rRNA gene sequences of the ten study strains (Table 1) and three *Microbacterium* type strains determined in this study and those of other 129 *Microbacterium* species. The 16S rRNA gene tree (Figure 1) showed that all the study strains were closely related to the type strains of seven *Microbacterium* species (*M. algeriense, M. liquefaciens, M. maritypicum, M. luteolum M. oxydans, M. paraoxydans*, and *M. saperdae*), most of which have been isolated from various habitats different from the marine environments selected in this study. The study strains were distributed into five groups: Group A contained strains KSW4-10^T^, KSW4-16, and SSW1-7 (with 100% sequence identity to one another) and was positioned between *M. algeriense*, isolated from oil production waters (Lenchi et al., 2020), and a clade including *M. liquefaciens* isolated from dairy products (Collins et al., 1983; Takeuchi and Hatano, 1998b), *M. maritypicum* from sea water and marine mud (ZoBell and Upham, 1944; Takeuchi and Hatano, 1998a), and *M. oxydans* from hospital materials (Chatelain and Second, 1966; Schumann et al., 1999); Group B consisted of only one isolate, SSW1-49^T^, and occupied a position between *M. saperdae* from dead larvae of an insect (Lysenko, 1959; Takeuchi and Hatano, 1998b) and *M. algeriense*; Group C encompassed strains KSW2-24^T^, KSW4-6, and SSW1-36 (intra-group sequence identity of 100%) and formed a distinct clade between *M. luteolum* from soil (Yokota et al., 1993b; Takeuchi and Hatano, 1998b) and *M. saperdae*; and Group D contained strains SSW1-47^T^ and SSW1-51 (with 100% sequence identity and the same origin of isolation) and were closely related to Group E encompassing strain KSW4-4 and *M. paraoxydans* isolated from human blood (Laffineur et al., 2003), sharing 100% sequence identity to each other (Figure 1, Supplementary Table S2).

Inter-group sequence similarities of the study strains ranged from 99.43% (8 nt differences) to 99.86% (2 nt differences), while the 16S rRNA gene sequence similarity values between the study strains and seven closely related type strains were 99.36% (9 nt differences)−100%. On the other hand, the above type strains showed sequence similarities between 99.38% (9 nt differences) and 100% to one another. Among them, the type strains of *M. liquefaciens*, M. *maritypicum*, and *M. oxydans* shared 100% sequence identity to one another and also showed high sequence similarity values (99.79–99.87%; 2–3 nt differences) with *M. algeriense, M. luteolum*, and *M. saperdae* (Supplementary Table S2). As visualized by very short lengths of branches in the 16S rRNA gene tree (Figure 1), these results indicate that the study strains and reference type strains have very close phylogenetic relationships with one another.

In addition, several pairs of *Microbacterium* species were also found to have very short lengths of branches in the 16S rRNA gene tree (Figure 1), with high 16S RNA gene sequence similarity (99.50–100%) for each pair. These species pairs are discussed in detail below.

### UBCG phylogenomic analysis

**Figure 2:**
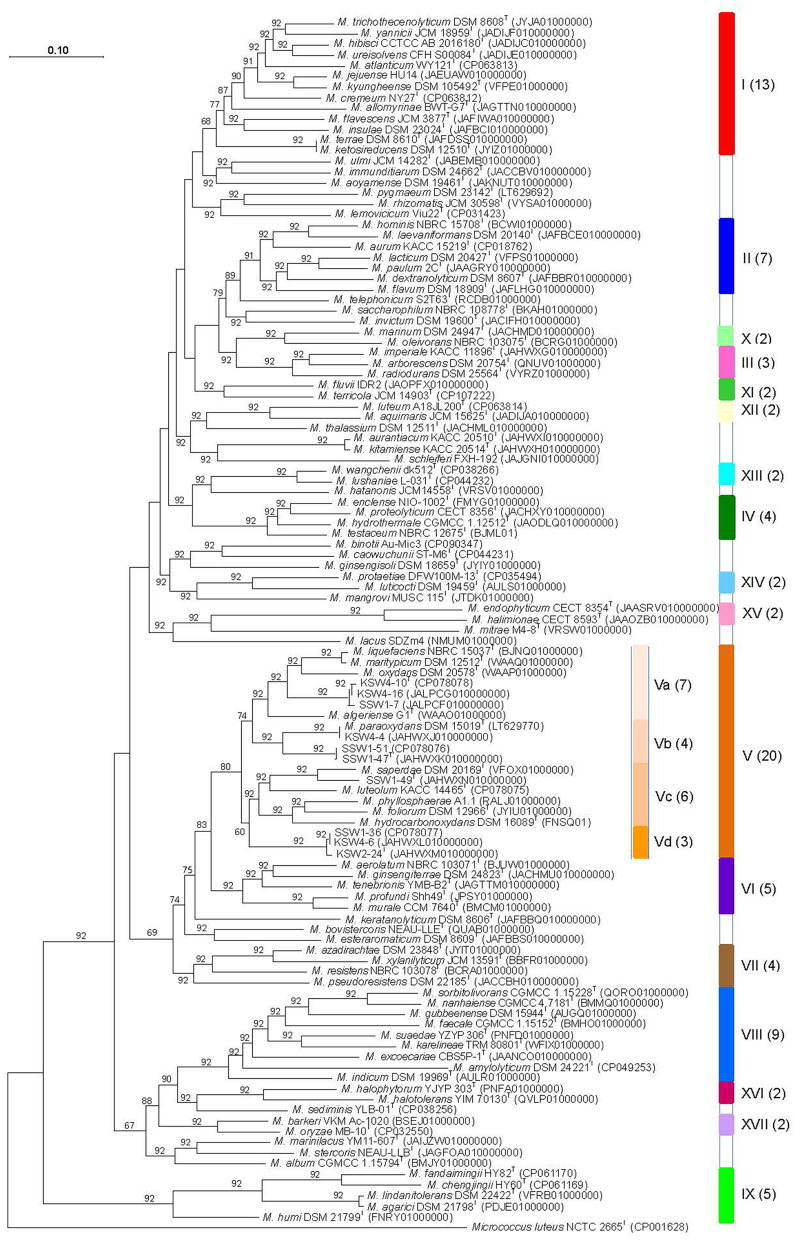
Core genome-based phylogenetic tree based on the genomes of the 10 study strains and 103 *Microbacterium* strains including 96 type and seven non-type strains. Colors on the right represent the clusters, most of which were defined at GSI of 92 and the mean intra-cluster OrthoANIu >80.00%. GSI values are given at branches.

The phylogenomic analysis was carried out based on 92 bacterial core gene sequences using the UBCG pipeline (Na et al., 2018), with the genomes of the ten study strains and 103 *Microbacterium* strains including 96 type and seven non-type strains. In the core genome-based phylogenetic tree (Figure 2), all the *Microbacterium* strains were resolved into nine major clusters (clusters I–IX including ≥three type strains), and eight minor clusters (clusters X–XVII with two type strains) were defined mostly at GSI of 92 and mean intra-cluster OrthoANIu of >80.00% (Table 2). The topologies of the nine major clusters were maintained irrespective of the addition/deletion of the related sequences and also supported in the core genome analyses reported previously (Dong et al., 2020; Bellassi et al., 2021; Tian et al., 2021). The origin of isolation for each type strain included in the clusters is given in Supplementary Table S3.

**Table 2:** Intra-cluster OrthoANIu values and DNA G+C contents of the nine major and eight minor clusters defined in genus *Microbacterium*.

**Cluster**	**Sub-cluster**	**OrthoANIu (%)**			**DNA G**+**C contents (%)**	
		**Mean**	**Minimum**	**Maximum**	**Minimum**	**Maximum**
I		81.23	79.17	99.97	69.0	70.9
II		81.04	79.20	84.40	69.4	71.0
III		82.82	81.97	84.24	69.0	70.8
IV		84.66	83.78	86.02	69.7	71.0
V		82.83	80.10	98.92	68.0	71.1
	Va	84.47	81.40	87.46	68.0	69.6
	Vb	84.02	82.67	84.51	70.0	71.1
	Vc	82.23	81.18	85.21	68.0	68.9
	Vd	98.09	97.92	98.42	69.2	69.4
VI		82.51	80.05	90.65	66.5	68.4
VII		81.19	78.32	88.47	68.3	71.2
VIII		78.10	74.52	85.76	65.4	71.8
IX		80.96	76.05	97.59	62.0	64.6
X		80.27			69.0	69.0
XI		80.53			70.4	70.4
XII		81.83			69.2	69.3
XIII		90.60			70.6	70.7
XIV		80.14			68.0	70.0
XV		84.85			61.6	61.8
XVI		80.05			68.0	70.8
XVII		83.87			71.1	71.4

Cluster I (*Microbacterium trichothecenolyticum* clade) consists of 13 species: *Microbacterium allomyrinae* (Lee and Kim, 2023), *Microbacterium atlanticum* and *Microbacterium cremeum* (Xie et al., 2021), *Microbacterium flavescens* (Collins et al., 1983; Takeuchi and Hatano, 1998b), *Microbacterium hibisci* (Yan et al., 2017), *Microbacterium insulae* (Yoon et al., 2009), *Microbacterium jejuense* (Kook et al., 2014), *Microbacterium kyungheense* (Kook et al., 2014), *Microbacterium ketosireducens* (Takeuchi and Hatano, 1998a), *M. terrae* and *M. trichothecenolyticum* (Yokota et al., 1993b; Takeuchi and Hatano, 1998b), *Microbacterium ureisolvens* (Cheng et al., 2019), and *Microbacterium yannicii* (Karojet et al., 2012). The cluster was defined relatively at low GSI (68) and showed intra-cluster OrthoANIu values of ≥79.17% (mean, 81.23%) and DNA G+C contents of 69.0–70.9% (Table 2). Most of the type strains also formed a coherent cluster in a previous core genome analysis (Xie et al., 2021), albeit that many of them formed well-separated sublines in the 16S rRNA gene trees (Dong et al., 2020; Lee and Kim, 2023; Figure 1). Among the species of which the genome sequences were not available yet, *M. arthrosphaerae* (Kämpfer et al., 2011) and *Microbacterium aureliae* were shown to be closely related to *M. atlanticum, M. hibisci, M. ureisolvens*, and *M. yannicii* in the 16S rRNA gene trees, albeit with low bootstrap support (Figure 1; Dong et al., 2020; Lee and Kim, 2023).

Cluster II (*Microbacterium lacticum* clade) contains seven species, namely, *M. aurum* (Yokota et al., 1993a), *M. dextranolyticum* (Yokota et al., 1993a), *Microbacterium flavum* (Kageyama et al., 2007a), *Microbacterium hominis* (Takeuchi and Hatano, 1998a)*, Microbacterium laevaniformans* (Dias and Bhat, 1962; Collins et al., 1983), *M. lacticum* (Orla-Jensen, 1919; Approved Lists, 1980), and *Microbacterium paulum* (Bellassi et al., 2021), and revealed intra-cluster OrthoANIu values≥79.20% (mean, 81.04%) and DNA G+C contents of 69.4–71.0% (Table 2). Most of the type strains were also found as a single cluster in a previous core genome analysis (Bellassi et al., 2021), albeit that many of the strains formed well-separated sublines in the 16S rRNA gene trees (Dong et al., 2020; Lee and Kim, 2023; Figure 1).

Cluster III (*Microbacterium imperiale* clade) is composed of *M. arborescens* (Imai et al., 1984), *M. imperiale* (Collins et al., 1983), and *Microbacterium radiodurans* (Zhang et al., 2010), with intra-cluster OrthoANIu values of 81.97–84.24% (mean, 82.82%) and DNA G+C contents of 69.0–70.8% (Table 2), and formed a coherent cluster in the 16S rRNA gene trees, with high bootstrap support (Dong et al., 2020; Lee and Kim, 2023; Figure 1) and the previous analyses of core genomes (Dong et al., 2020; Bellassi et al., 2021; Tian et al., 2021).

Cluster IV (*Microbacterium testaceum* clade) consists of four species, namely, *Microbacterium enclense* (Mawlankar et al., 2015), *M. hydrothermale* (Zhang et al., 2014), *Microbacterium proteolyticum* (Alves et al., 2015), and *M. testaceum* (Takeuchi and Hatano, 1998b), the type strains of which shared intra-cluster OrthoANIu values of 83.78–86.02% (mean, 84.66%) and DNA G+C contents of 69.7–71.0% (Table 2). Most of them were also shown to be clustered together in the core genome-based phylogenetic trees reported previously (Dong et al., 2020; Tian et al., 2021; Xie et al., 2021). Although the genome sequence are not determined yet, *Microbacterium zeae* (Gao et al., 2017) was tightly associated with *M. enclense* and *M. proteolyticum* of this cluster in the 16S rRNA gene trees (Dong et al., 2020; Lee and Kim, 2023; Figure 1).

Cluster V (*Microbacterium liquefaciens* clade) is the largest and encompasses 10 *Microbacterium* species and the five groups of the study strains defined in 16S rRNA gene tree (Figure 1). This clade was defined at GSI of 80 and showed intra-cluster OrthoANIu values of ≥80.10% (mean, 82.83%) and DNA G+C contents of 68.0–71.1%, the strains of which were further assigned to four subclusters (Va–Vd) with support of 92 GSI. Subcluster Va contains the type strains of *M. algeriense, M. liquefaciens, M. maritypicum*, and *M. oxydans* together with three study strains, KSW4-10^T^, KSW4-16, and SSW1-7, having intra-subcluster OrthoANIu values of 81.40–87.46% (mean, 84.87%) and DNA G+C contents of 68.0–69.6%; subcluster Vb is composed of the type strain of *M. paraoxydans* and three study strains, KSW4-4, SSW1-47^T^, and SSW1-51, with intra-subcluster OrthoANIu values of 82.67–84.51% (mean, 84.02%) and DNA G+C contents of 70.0–71.1%; subcluster Vc encompasses the five type strains of *M. luteolum, M. saperdae, M. foliorum*, and *M. phyllosphaerae* (Behrendt et al., 2001), and *M. hydrocarbonoxydans* (Schippers et al., 2005), and one study strain, SSW1-49^T^, sharing intra-subcluster OrthoANIu values of 81.18–85.21% (mean, 82.23%) and DNA G+C contents of 68.0–68.9%; and subcluster Vd includes only the study strains, KSW2-24^T^, KSW4-6, and SSW1-36, with intra-subcluster OrthoANIu values of 97.92–98.42% (mean, 98.09%) and DNA G+C contents of 69.2–69.4% (Table 2). Among them, the six or seven type strains were associated together in the previous analyses of core genomes (Bellassi et al., 2021; Tian et al., 2021; Xie et al., 2021). In addition, most of the type strains were closely associated with one another in the 16S rRNA gene trees, while three species (*M. foliorum* and *M. phyllosphaerae*, and *M. hydrocarbonoxydans*) formed well-separated sublines from the other type strains (Dong et al., 2020; Lee and Kim, 2023; Figure 1).

Cluster VI (*Microbacterium aerolatum* clade) is composed of five species, namely, *M. aerolatum* (Zlamala et al., 2002), *Microbacterium ginsengiterrae* (Kim et al., 2010), *M. murale* (Kämpfer et al., 2012), *Microbacterium profundi* (Wu et al., 2008), and *Microbacterium tenebrionis* (Lee and Kim, 2023), and showed intra-cluster OrthoANIu values of 80.05–90.65% (mean, 82.51%) and DNA G+C contents of 66.5–68.4% (Table 2). The recently described *Microbacterium ihumii* (Yacouba et al., 2022) was also included in this cluster (data not shown). All the type strains also formed a single cluster in the previous analyses of core genomes (Lee and Kim, 2023) and whole genomes (Yacouba et al., 2022), although they were divided into two lineages in the 16S rRNA gene trees (Figure 1; Lee and Kim, 2023). Among the species of which the genome sequences were not determined yet, *Microbacterium panaciterrae* (Nguyen et al., 2015), *Microbacterium shaanxiense* (Peng et al., 2015), and *Microbacterium tumbae* (Nishijima et al., 2017) formed a coherent cluster with *M. aerolatum, M. ginsengiterrae*, and *M. tenebrionis* of this cluster in the 16S rRNA gene tree (Figure 1).

Cluster VII (*Microbacterium resistens* clade) consists of four species, namely, *Microbacterium azadirachtae* (Madhaiyan et al., 2010), *Microbacterium pseudoresistens* (Young et al., 2010), *Microbacterium resistens* (Behrendt et al., 2001), and *Microbacterium xylanilyticum* (Kim et al., 2005), the type strains of which showed intra-cluster OrthoANIu values of 78.32–88.47% (mean, 81.19%) and DNA G+C contents of 68.3–71.2% (Table 2). Members of this cluster were also associated together in the previous phylogenetic trees based on core genomes (Dong et al., 2020; Lee and Kim, 2023), albeit with the formation of four independent sublines in the 16S rRNA gene trees (Figure 1; Lee and Kim, 2023).

Cluster VIII (*Microbacterium gubbeenense* clade) was defined at GSI of 92 and contains nine type species, forming a tight cluster with high bootstrap support in the 16S rRNA gene trees (Dong et al., 2020; Lee and Kim, 2023; Figure 1): *M. amylolyticum* (Anand et al., 2012), *Microbacterium excoecariae* (Chen et al., 2020), *Microbacterium faecale* (Chen et al., 2016), *M. gubbeenense* (Brennan et al., 2001), *Microbacterium indicum* (Shivaji et al., 2007), *Microbacterium karelineae* (Zhu et al., 2021), *Microbacterium nanhaiense* (Yan et al., 2015), *Microbacterium sorbitolivorans* (Meng et al., 2016), and *M. suaedae* (Zhu et al., 2019). The intra-cluster OrthoANIu values and DNA G+C contents were 74.52–85.76% (mean, 78.10%) and 65.4–71.8%, respectively (Table 2). All the type strains formed a tight cluster with high bootstrap support in the 16S rRNA gene tree (Figure 1) and were also found as a single cluster in our previous core genome-based phylogenetic tree (Lee and Kim, 2023).

Cluster IX (*Microbacterium agarici* clade) forms the deepest branch within the genus *Microbacterium* and contains the five type strains of *M. agarici* and *Microbacterium humi* (Young et al., 2010), *Microbacterium lindanitolerans* (Lal et al., 2010), and *Microbacterium chengjingii* and *Microbacterium fandaimingii* (Zhou et al., 2021), being well separated from the other *Microbacterium* species with support of 92 GSI. This fact was also supported in both the previous core genome-based phylogenetic trees (Bellassi et al., 2021; Lee and Kim, 2023) and 16S rRNA gene trees (Figure 1; Lee and Kim, 2023). The intra-cluster OrthoANIu values were 76.05–97.59% (mean, 80.96%) and DNA G+C contents of 62.0–64.6% (Table 2).

Among the eight minor clusters (X–XVII) shown in Figure 2, *Microbacterium aquimaris* Kim et al. 2008–*Microbacterium luteum* Xie et al. 2021 (Cluster XII; 81.83% OrthoANIu), *Microbacterium endophyticum* Alves et al. 2014 –*Microbacterium halimionae* Alves et al. 2014 (Cluster XV; 84.85% OrthoANIu), *Microbacterium protaetiae* Heo et al. 2020–*Microbacterium luticocti* (Vaz-Moreira et al., 2008) (Cluster XIV; 80.14% OrthoANIu), *Microbacterium halotolerans* (Li et al., 2005)–*Microbacterium halophytorum* (Li et al., 2018) (Cluster XVI; 80.05% OrthoANIu), and *Microbacterium barkeri* Takeuchi and Hatano 1998b–*Microbacterium oryzae* (Kumari et al., 2013) (Cluster XVII; 83.37% OrthoANIu) pairs (Table 2) are also well supported by the 16S rRNA gene trees (Dong et al., 2020; Lee and Kim, 2023; Figure 1) and the previous analyses of core genomes (Dong et al., 2020; Bellassi et al., 2021; Tian et al., 2021; Xie et al., 2021; Lee and Kim, 2023). On the other hand, the remaining three minor clusters, namely, *Microbacterium marinum* Zhang et al. 2012–*M. oleivorans* Schippers et al. 2005 (Cluster X; 80.27% OrthoANIu), *Microbacterium fluvii* (Kageyama et al., 2007c)–*Microbacterium terricola* (Kageyama et al., 2007b) (Cluster XI; 80.53% OrthoANIu), and *Microbacterium wangchenii* Dong et al. 2020 –*Microbacterium lushaniae* Tian et al. 2021 pairs (Cluster XIII; 90.60% OrthoANIu), formed tight clades in the core genome-based phylogenetic tree (Figure 2, Table 2), but each type strain occupied independent positions in the 16S rRNA gene trees (Figure 1; Dong et al., 2020; Lee and Kim, 2023).

A further 27 *Microbacterium* strains constitute single-membered clusters, most of which showed low OrthoANIu values < 80.00% with members of the defined clusters above, albeit with support by high GSI in the core genome-based phylogenetic tree (Figure 2).

### OGRI analyses

The OrthoANIu and dDDH values between the study strains and closely related type strains are given in Supplementary Table S3. The study strains which were defined as five groups in the 16S rRNA gene tree (Figure 1) were found to belong to the cluster V (*M. liquefaciens clade*) in the core genome-based phylogenomic tree (Figure 2). The intra-group OrthoANIu values of the groups A (KSW4-10^T^, KSW4-16, and SSW1-7), C (KSW2-24^T^, KSW4-6, and SSW1-36), and D (SSW1-47^T^ and SSW1-51) were 97.74–98.89%, 97.92–98.42%, and 98.9%, respectively, while the inter-group OrthoANIu values were relatively low and ranged from 80.90 to 82.67% (Supplementary Table S4), revealing that the above three groups constituted independent taxa (Richter and Roselló-Móra, 2009). On the other hand, the strains of the group A showed high OrthoANIu values with the type strains of *M. liquefaciens* and *M. maritypicum* (85.31–85.64% and 85.64–85.90%, respectively). Strain SSW1-49^T^ (group B) and the strains of the group C revealed high OrthoANIu values with the type strain of *M. saperdae* (89.40% and 80.80–81.95%, respectively), whereas the strains of the group D and KSW4-4 (group E) revealed high OrthoANIu values (84.40–84.48% and 98.9%, respectively) with the type strain of *M. paraoxydans* (Table 3). Concerning the ANI threshold (95–96%) for delineation of species (Richter and Roselló-Móra, 2009), these results support that the groups A–D represent four new species of the genus *Microbacterium* and strain KSW4-4 is a strain of *M. paraoxydans*, albeit with different origin of isolation.

**Table 3:** OrthoANIu and dDDH values calculated between the 10 study strains and most closely related type strains.

**Group^a^**	**Strain(s)**	**Most closely related type strain(s)**	**OrthoANIu (%)**	**dDDH (%)**
A	KSW4-10^T^ KSW4-16 SSW1-7	*M. maritypicum* DSM 12512^T^	85.64–85.90	29.5–29.8
		*M. liquefaciens* NBRC 15037^T^	85.31–85.64	29.2–29.3
B	SSW1-49^T^	*M. saperdae* DSM 20169^T^	89.4	37.5
C	KSW2-24^T^ KSW4-6 SSW1-36	*M. saperdae* DSM 20169^T^	81.83–81.95	24.4–24.5
D	SSW1-47^T^ SSW1-51	*M. paraoxydans* DSM 15019^T^	84.40–84.48	27.9–28.0
E	KSW4-4	*M. paraoxydans* DSM 15019^T^	98.90	91.3

^a^defined in the 16S rRNA gene tree.

The calculation of dDDH values also supported the results of ANI analysis. The intra-group dDDH values of the groups A, C, and D were 80.6–91.2%, 81.8–85.9%, and 90.9%, respectively, being higher than the threshold (70%) for prokaryotic species delineation (Wayne et al., 1987), while the inter-group dDDH values of the groups A–D were relatively low ( ≤ 25.0%) (Supplementary Table S4). On the other hand, all the study strains showed low dDDH values ( ≤ 37.5%) with the seven closely related type strains, with the exception that strain KSW4-4 shared a high dDDH (91.3%) with the type strain of *M. paraoxydans* (Table 3).

### Phenotypic characteristics

The results for a total of 91 physiological and biochemical tests were compared for the study strains and seven closely related type strains. Among them, 44 phenotypic traits (48.4%) were positive or negative for all of the test strains (Supplementary Table S5). Differential phenotypic characteristics among the test strains contained 21 acid productions from substrates, seven carbon source assimilations, 14 enzyme activities, pH and temperature ranges for growth, and NaCl tolerance (Supplementary Table S6). The strains of the group A showed the largest intra-group variation (4.4%) and were variable in acid production from D-melezitose, D-melibiose, L-rhamnose, and ribose according to the strains. The strains of the groups C and D were variable in acid production from N-acetyl-glucosamine. In addition, gelatinase activity was also variable in the strains of the group C (Supplementary Table S6).

In some of the type strains tested in this study, there were shown to be the phenotypic traits that conflict with the previously described results (Collins et al., 1983; Takeuchi and Hatano, 1998a; Schumann et al., 1999; Laffineur et al., 2003; Lenchi et al., 2020). All the physiological and biochemical characters of the type strains were examined under the same conditions with the study strains except that acid production was recorded after 7-day incubation. *M. paraoxydans* KACC 14506^T^ revealed the largest difference (12.1%) from the results of Laffineur et al. (2003) for up to 11 phenotypic traits (acid production from five substrates, four enzyme activities, and growth at 10°C and 42°C). *M. maritypicum* KACC 14436^T^ showed the results (5.5%) conflicting with data of Takeuchi and Hatano (1998a) in one assimilation and four acid production tests. *M. algeriense* DSM 109018^T^ (5.5%) differed from data of Lenchi et al. (2020) in growth tests at 10°C, 50°C, and pH 5, together with glucose fermentation and assimilation of L-arabinose, while *M. liquefaciens* KACC 14464^T^ and *M. oxydans* KACC 14467^T^ differed from the results reported previously (Collins et al., 1983; Schumann et al., 1999) only in one or two phenotypic traits (Supplementary Tables S5, S6), respectively.

The study strains contained D-ornithine as the diagnostic diamino acid and N-glycolylated mureins in their cell walls, a polar lipid profile including diphosphatidylglycerol, phosphatidylglycerol, and an unidentified glycolipid; and the fully unsaturated menaquinones with 10, 11, and 12 isoprene units (MK-10, MK-11, and MK-12, respectively). The type strains of their phylogenetically close neighbors showed the same patterns of diamino acid in the cell walls and polar lipids but did not contain MK-10 that was detected in the study strains (Supplementary Table S7). The cellular fatty acids of all the study strains and the seven reference type strains consisted mainly of *iso*- and *anteiso*-branched components (Supplementary Table S8). Most of the strains contained *antiso*-C_15:0_ (36.6–68.1%), *antiso*-C_17:0_ (13.7–33.7%), and *iso*-C_16:0_ (10.6–25.4%) as the major fatty acids (>10% of the total). The study strains of the group A and *M. luteolum* KACC 14465^T^ also contained *iso*-C_15:0_ (13.6–15.8%), *iso*-C_15:0_ (12.1%), and *anteiso*-C_15:1_ A (10.6%), respectively, as additional major fatty acids, while *M. liquefaciens* KACC 14464^T^ and *M. maritypicum* KACC 14436^T^ contained small amounts of *iso*-C_16:0_ (9.4%) and *antiso*-C_17:0_ (8.1%), respectively, as compared with all the other strains (Supplementary Table S8).

### Reclassification of *Microbacterium* species

#### *Microbacterium ketosireducens* Takeuchi and Hatano 1998 is a later heterotypic synonym of *Microbacterium terrae* (Yokota et al., 1993) Takeuchi and Hatano 1998

*M. ketosireducens* IFO 14548^T^ (Takeuchi and Hatano, 1998a) isolated from soil is known to have an ability to reduce 2,5-diketo-D-gluconic acid to 2-keto-D-gluconic acid. In the same year, *M. terrae* (Takeuchi and Hatano, 1998b) was assigned to the redefined genus *Microbacterium* from *Aureobacterium terrae* (Yokota et al., 1993b) through the union of the genera *Microbacterium* and *Aureobacterium* based on 16S rRNA gene phylogeny. In the original description, *M. ketosireducens* IFO 14548^T^ was shown to share *in vitro* DDH value of 47% with *M. terrae* IFO 15300^T^, but the 16S rRNA gene sequence similarity between both of the type strains was not compared.

In this study, *M. ketosireducens* IFO 14548^T^ *and M. terrae* IFO 15300^T^ were found to show 16S rRNA gene sequence similarity of 99.72% (4 nt differences) to each other, both of which sequences revealed 99.65% (5 nt differences) with their genome-derived ones, respectively (Supplementary Table S1), while the 16S rRNA gene sequence (MZ433298) of *M. terrae* KACC 14470^T^ determined in this study shared 100% sequence identity with the genome-derived ones of *M. terrae* DSM 8610^T^ (JAFDDS010000001) and *M. ketosireducens* DSM 12510^T^ (JYIZ01000009). The OGRI analysis indicated that both of the type strains shared a dDDH value of 100% and an OrthoANIu of 99.97% to each other (Table 4), supporting the conclusion that *Microbacterium ketosireducens* Takeuchi and Hatano 1998 is a later heterotypic synonym of *Microbacterium terrae* (Yokota et al. 1993) Takeuchi and Hatano 1998 according to the page priority [Rule 24b (4) of the International Code of Nomenclature of Prokaryotes (ICNP; Oren et al., 2022)], for names validly published in the same year.

**Table 4:** Species that are reclassified as later heterotypic synonyms of preexisting species.

**Synonym**	**Species**	**16S rRNA gene sequence similarity (%)**	**OrthoANIu (%)**	**dDDH (%)**
*Microbacterium ketosireducens* Takeuchi and Hatano 1998	*Microbacterium terrae* (Yokota et al., 1993) Takeuchi and Hatano 1998	100	99.97	100
*Microbacterium kitamiense* Matsuyama et al. 1999	*Microbacterium aurantiacum* Takeuchi and Hatano 1998	100	97.89	81.9
*Microbacterium lindanitolerans* Lai et al. 2010	*Microbacterium agarici* Young et al. 2010	100	97.59	78.6
*Microbacterium maritypicum* (ZoBell and Upham, 1944) Takeuchi and Hatano 1998	*Microbacterium liquefaciens* (Collins et al., 1983) Takeuchi and Hatano 1998	100	98.30	84.3

#### *Microbacterium kitamiense* Matsuyama et al. 1999 as a later heterotypic synonym of *Microbacterium aurantiacum* Takeuchi and Hatano 1998

*M. kitamiense* Kitami C2^T^ (Matsuyama et al., 1999), a polysaccharide-producing bacterium isolated from the wastewater of a sugar-beet factory, was shown to be closely associated with the type strain of *M. aurantiacum* in the 16S rRNA gene tree (Figure 1). In that study, the *in vitro* DDH value between both of the type strains was 68%, with their DNA G+C contents of 69.2 mol% and 69.3 mol%, respectively, but 16S rRNA gene sequence similarity was not presented.

For the calculation of precise 16S rRNA gene sequence similarity and OGRI values, the genome sequences of *M. kitamiense* KACC 20514^T^ and *M. aurantiacum* KACC 20510^T^ were determined in this study. The genome-derived 16S rRNA gene sequence (JAHWXH010000003) of *M. kitamiense* KACC 20514^T^ was the same as the corresponding sequence (AB013919) of *M. kitamiense* Kitami C2^T^ (Supplementary Table S1), while the complete genome-derived 16S rRNA gene sequence of *M. aurantiacum* KACC 20510^T^ was obtained through concatenation of the two partial ones present in different contigs of the genome (JAHWXI01000000), showing 100% sequence identity with sequence EU863415 of *M. aurantiacum* CIP 105730^T^ determined by the Sanger method. Both of the type strains showed an OrthoANIu of 97.89% and a dDDH of 81.9% to each other (Table 4). Considering the thresholds for species delineation (Wayne et al., 1987; Richter and Roselló-Móra, 2009), *Microbacterium kitamiense* Matsuyama et al. 1999 should be considered as a later heterotypic synonym of *Microbacterium aurantiacum* Takeuchi and Hatano 1998.

#### *Microbacterium maritypicum* (ZoBell and Upham 1944) Takeuchi and Hatano 1998 is a later heterotypic synonym of *Microbacterium liquefaciens* (Collins et al. 1983) Takeuchi and Hatano 1998

*M. maritypicum* Takeuchi and Hatano 1998 was proposed by the reclassification of *Flavobacterium maritypicum* (ZoBell and Upham, 1944) based on phenotypic characteristics, together with *in vitro* DDH and 16S rRNA gene analysis, and shown to have *in vitro* DDH values of 13–27% to the close relatives (*M. liquefaciens, M. luteolum*, and *M. saperdae*), although there was no comparison of their 16S rRNA gene sequences. In the same year, *M. liquefaciens* (Takeuchi and Hatano, 1998b) was assigned to the redefined genus *Microbacterium* from *Aureobacterium liquefaciens* (Collins et al., 1983) through the union of the genera *Microbacterium* and *Aureobacterium* based on 16S rRNA gene phylogeny.

In this study, *M. maritypicum* belongs to cluster V (*M. liquefaciens* clade) in the core genome-based phylogenetic tree (Figure 2) and was closely related to six species (*M. algeriense, M. liquefaciens, M. luteolum, M. oxydans, M. paraoxydans*, and *M. saperdae*), as visualized by very short lengths of branches in the 16S rRNA gene tree (Figure 1). Comparison of all their genome-derived 16S rRNA gene sequences revealed that *M. maritypicum* DSM 12512^T^ shares 100% sequence identity with the type strains of *M. liquefaciens* and *M. oxydans* and shows sequence similarity of 99.45–99.87% (2–8 nt differences) with the remaining type strains (Supplementary Table S2). The OGRI analysis showed that *M. maritypicum* DSM 12512^T^ shares high OrthoANIu (98.30%) and dDDH (84.3%) values with *M. liquefaciens* NBRC 15037^T^ (Table 4, Supplementary Table S4), while its OrthoANIu (82.19–87.18%) and dDDH ( ≤ 32.1%) values with the remaining type strains were relatively low (Supplementary Table S4). Based on the OGRI thresholds for species demarcation (Wayne et al., 1987; Richter and Roselló-Móra, 2009), these results support the conclusion that *Microbacterium maritypicum* (ZoBell and Upham, 1944) Takeuchi and Hatano 1998 is a later heterotypic synonym of *Microbacterium liquefaciens* (Collins et al., 1983) Takeuchi and Hatano 1998 according to the page priority [Rule 24b (4) of the ICNP], for names validly published in the same year.

#### *Microbacterium* species maintained or needed to be further verified as separate species

Several type strain pairs with high 16S rRNA gene sequence similarity (≥99.72%; up to 3 nt differences) were found in 16S rRNA gene analysis. The *M. halimionae*–*M. endophyticum* pair (Alves et al. 2014) share high sequence similarity (99.8%; 3 nt differences) but showed the OrthoANIu 84.85% of and dDDH of 28.0% to each other (Table 5). *M. arborescens* was described by Imai et al. (1984) only based on phenotypic differences without genotypic analysis. In this study, *M. arborescens* DSM 20754^T^ shared 16S rRNA gene sequence similarity of 99.72% (3 nt differences) with *M. imperiale* KACC 11896^T^, but the OrthoANIu and dDDH values between both of the type strains were 84.24% and 27.2%, respectively (Table 5). In addition, *M. oxydans* DSM 20578^T^ shared 100% sequence identity in 16S rRNA gene with the type strains of *M. liquefaciens* and *M. maritypicum* (Supplementary Table S2), but it showed an OrthoANIu of 87.46% and a dDDH of 32.7% with *M. liquefaciens* NBRC 15037^T^, followed by an OrthoANIu of 87.18% and a dDDH of 32.1% with *M. maritypicum* DSM 12512^T^ (Table 5). These results of the OGRI analysis support their original descriptions of the above six species as separate taxa (Wayne et al., 1987; Richter and Roselló-Móra, 2009), except for *M. maritypicum* (see above).

**Table 5:** Species that are maintained or needed to be further verified as separate species.

**Species**	**Species**	**16S rRNA gene sequence similarity (%)**	**OrthoANIu (%)**	**dDDH (%)**
*Microbacterium halimionae* Alves et al. 2014	*Microbacterium endophyticum* Alves et al. 2014	99.79 (3 nt)	84.85	28.0
*Microbacterium imperiale* (Steinhaus, 1941) Collins et al. 1983	*Microbacterium arborescens* (ex Frankland and Frankland, 1889) (Imai et al., 1984)	99.72 (3 nt)	84.24	27.2
*Microbacterium oxydans* (Chatelain and Second, 1966) Schumann et al. 1999	*Microbacterium liquefaciens* (Collins et al., 1983) Takeuchi and Hatano 1998	100	87.46	32.7
	*Microbacterium maritypicum* (ZoBell and Upham, 1944) Takeuchi and Hatano 1998	100	87.18	32.1
*Microbacterium neimengense* Gao et al. 2013	*Microbacterium binotii* Clermont et al. 2009	99.86 (2 nt)	ND	ND
*Microbacterium pumilum* Kageyama et al. 2006	*Microbacterium deminutum* Kageyama et al. 2006	99.79 (3 nt)	ND	ND

Data in parentheses represent the number of nucleotide differences. ND, not determined because the genomes were not available.

In addition, high 16S rRNA gene sequence similarity (> 99.79%; 3 nt differences) was found for two other species pairs, *M. deminutum*–*M. pumilum* (Kageyama et al., 2006) and *M. neimengense* (Gao et al., 2013)–*M. biotini* (Clermont et al., 2009) (Table 5), as revealed by very short lengths of branches in 16S rRNA gene phylogeny (Figure 1), but their genome sequences are not available yet. Considering that *in vitro* DDH assays for some *Microbacterium* type strains were inaccurate (Takeuchi and Hatano, 1998a; Matsuyama et al., 1999), the genome sequencing and analysis of these four species are further required.

### Transfer of five *Microbacterium* species to a new genus

The five type strains of cluster IX (*M. agarici* clade) that form the deepest branch in the phylogenomic tree (Figure 2) are shown to be well separated from the other *Microbacterium* species, which is also supported in 16S rRNA gene trees (Dong et al., 2020; Bellassi et al., 2021; Tian et al., 2021; Lee and Kim, 2023; Figure 1). To determine the generic assignment of these species (*M. agarici, M. chengjingii, M. fandaimingii, M. humi*, and *M. lindanitolerans*), AAI values were determined between the five type strains of these species and all the other *Microbacterium* strains. As a result, the intra-cluster AAI values of cluster IX were ≥72.64% (mean, 80.95%), while the inter-cluster AAI values with other members of the genus *Microbacterium* were ≤ 59.18% (mean, 57.27%) (Table 6, Supplementary Table S9), being notably lower than the thresholds (74–76% or 68%) proposed for genus delineation (Wirth and Whitman, 2018; Nicholson et al., 2020; Zheng et al., 2020). These results support the conclusion that the five type strains of cluster IX should be transferred to a new genus of the family *Microbacteriaceae* separated from the genus *Microbacterium*, for which the name *Paramicrobacterium* gen. nov. is proposed. Among these species, *M. agarici* (Young et al., 2010) and *M. lindanitolerans* (Lal et al., 2010) were not compared to each other for their 16S rRNA gene sequences because they were published in the same year. In this study, both of the type strains were shown to share 16S rRNA gene sequence similarity of 100%, an OrthoANIu of 97.59 %, and a dDDH of 78.6% to each other (Table 4), supporting the conclusion that *Microbacterium lindanitolerans* Lal et al. 2010 is a later heterotypic synonym of *M. agarici* Young et al. 2010 according to the page priority [Rule 24b (4) of the ICNP], for names validly published in the same year.

**Table 6:** Intra- and inter-cluster AAI values (%) of cluster IX (*Microbacterium agarici* clade) in the genus *Microbacterium*.

	**Mean**	**Minimum**	**Maximum**
Intra-cluster IX (*Microbacterium agarici* clade)	80.95	72.64	97.88
Inter-cluster IX (*Microbacterium agarici* clade)	57.27	55.30	59.18

## Discussion

The 16S rRNA gene has traditionally been used as a good molecular marker for taxonomic studies, albeit with the limitation in resolving phylogenetic relationships at the species level (Carro et al., 2018; Na et al., 2018). In this study, before performing 16S rRNA gene-based phylogenetic analysis, the 16S rRNA gene sequences available for each type strain of all *Microbacterium* species were retrieved from public databases, such as the LPSN, EzBioCloud, and NCBI, and compared for checking their authenticity and completeness. As a result, it was shown that many of the 16S rRNA gene sequences were slightly inaccurate when compared with genome-derived sequences, while some of them were incorrectly reported as the reference sequence of the type strains in LPSN. The sequences of *M. hydrothermale* 0704CP-2^T^ (HM222660), *M. rhizosphaerae* CHO1^T^ (KP72259), and *M. suaedae* YZYP 306^T^ (MF084212) listed in their original descriptions and LPSN, together with those of *M. aerolatum* CCM 4955^T^ (MT760116) and *M. dextranolyticum* IFO 14592^T^ (D21341 and AB007417) contained in NCBI, revealed sequence similarities of 98.50–99.51% (7–21 nt differences) compared with the original or corresponding genome-derived ones. Considering that some *Microbacterium* type strains, albeit with revealing very short branch lengths in the 16S rRNA gene tree, are maintained as independent taxa at the species level, it is evident that the use of the above six sequences in the 16S rRNA gene phylogeny can make it very difficult to unravel their intrinsic phylogenetic relationships. Moreover, the 16S rRNA gene sequences of *M. amylolyticum* CCM 4955^T^ (MT760185) and *M. arthrosphaerae* CCM 7681^T^ (MT760166) listed in LPSN and *M. testaceum* CCM 2299^T^ (MT760091) contained in NCBI were found to be the same with those given in the original descriptions of the type strains of *M. arthrosphaerae, M. murale*, and *M. amylolyticum*, respectively. These results indicate that the MT760185 and MT760166 were incorrectly listed as the reference sequences of *M. amylolyticum* and *M. arthrosphaerae*, respectively, in LPSN.

The 10 strains isolated from seaweeds from two beaches (Table 1), together with *M. arthrosphaerae* KACC 16680^T^, *M. luteolum* KACC 14465^T^, and *M. terrae* KACC 14470^T^, were subjected to 16S rRNA gene sequencing to identify their closely related neighbors. The 16S rRNA gene analysis with near full-length sequences showed that the strains from this study can be divided into five groups with 100% intra-group sequence identity. Very close phylogenetic relationships were found among the study strains and between the study strains and reference type strains of *M. algeriense, M. liquefaciens, M. luteolum, M. maritypicum, M. oxydans, M. paraoxydans*, and *M. saperdae*, as given by the very short lengths of the tree branches and high 16S RNA gene sequence similarity (99.36–100%; 0–9 nt differences). Among the type strains, *M. liquefaciens* NBRC 15037^T^, *M. maritypicum* DSM 12512^T^, and *M. oxydans* DSM 20578^T^ share 100% identity in 16S rRNA gene sequence to one another.

In this study, we report the overall phylogenomic clustering of the genus *Microbacterium* by core genome analysis, with a total of the 113 genomes, including the ten study strains and 103 reference *Microbacterium* strains. In general, core genome-based phylogenetic trees are known to be consistent with those using whole-genome data (Riesco et al., 2018) and can additionally provide bootstrap support by means of GSI values. The core genome-based phylogenetic tree reveals that nine major, eight minor, and 27 single-membered clusters can be defined, most of which were established at GSI of 92 and mean intra-cluster OrthoANIu values of >80.00%. Considering that the genome sequences of 28 *Microbacterium* type strains are not still available yet, the presence of many single-membered clusters suggests that the genus *Microbacterium* is still under-speciated. Only three of the major clusters were recognized in their entirety in the 16S rRNA gene trees: the clusters III (*M. imperiale* clade), VIII (*M. gubbeenense* clade), and IX (*M. agarici* clade), although *M. amylolyticum* of cluster VIII showed significantly lower OrthoANIu values (75.40–75.78%) and DNA G+C content (65.4%) than the mean intra-cluster OrthoANIu value (78.10%) and DNA G+C content (68.9%). The five species of cluster IX consistently formed the deepest branches in both the core genome- and 16S rRNA gene-based phylogenetic trees. Moreover, they formed a distinct cluster at a position located remotely from members of the genus *Microbacterium* in the whole-genome-based phylogenetic tree of the class *Actinobacteri*a (Nouioui et al., 2018). This overall phylogenomic clustering of the genus *Microbacterium* can provide a framework for the future descriptions of new taxa. Despite the recent increase of available genome sequences, the overall phylogenomic analysis based on whole- or core genome sequences for the genus *Microbacterium* was not performed to date. In the recent descriptions of new species (Dong et al., 2020; Bellassi et al., 2021; Tian et al., 2021; Xie et al., 2021), core genome-based phylogenetic analyses have been partially performed with the 26–49 type strains of the genus *Microbacterium*. Among the *Microbacterium* strains included in the previous phylogenomic analyses (Dong et al., 2020; Tian et al., 2021), the genomes of “*Microbacterium barkeri*” 2011-R4 (AKVP01000000), “*Microbacterium chocolatum*” SIT 101 (CP015810), “*M. kitamiense*” Sa12 (PGGU01000000), and “*Microbacterium paludicola*” CC3 (CP018134) were not those of the type strains of the corresponding species in that the genome-derived 16S rRNA gene sequences were considerably different from those of the type strains determined by Sanger method, indicating that they should not be used as reference genomes.

The OGRI represent any measurements indicating how similar are two genome sequences (Chun and Rainey, 2014). Among them, ANI and dDDH have been widely used for prokaryotic species demarcation. The ANI threshold (95–96%) proposed for species delineation has been found to be correlated with the *in vitro* DDH threshold of 70% (Goris et al., 2007; Richter and Roselló-Móra, 2009). It is now evident that the dDDH calculated through comparisons of whole-genome sequences is more unbiased for discriminating between closely related strains than the values determined by error-prone and labor-extensive DDH experiments (Rosselló-Móra et al., 2011; Meier-Kolthoff et al., 2013). In this study, the dDDH values calculated from the genomes of some type strain pairs were found to be very higher than those determined by *in vitro* DDH experiments in previous studies (Takeuchi and Hatano, 1998a; Matsuyama et al., 1999). The results of the OGRI analysis support that the strains of the four groups (A–D) represent four new species of the genus *Microbacterium*.

The dDDH values estimated from the genomes of six pairs of the closely related type strains showed that three fell above the 70% threshold (Wayne et al., 1987), indicating that *M. ketosireducens* (Takeuchi and Hatano 1998a), *M. kitamiense* (Matsuyama et al. 1999), and *M. maritypicum* (Takeuchi and Hatano 1998a) are later heterotypic synonyms of preexisting species, while the others fell below the 70% threshold (Wayne et al., 1987), supporting that *M. halimionae* and *M. endophyticum* (Alves et al. 2015), *M. imperiale* (Collins et al., 1983) and *M. arborescens* (Imai et al., 1984), and *M. oxydans* (Schumann et al. 1999) and *M. liquefaciens* (Takeuchi and Hatano 1998b) are appropriately classified as distinct species.

Furthermore, AAI values have been used to define the genus boundary in several families (Wirth and Whitman, 2018; Nicholson et al., 2020; Zheng et al., 2020), although a standardized threshold has not been established. In this study, members of cluster IX (*M. agarici* clade) showed significantly lower inter-cluster AAI values with other members of the genus *Microbacterium* than the thresholds proposed for genus delineation. The results of AAI analysis, together with distinctness in all the phylogenetic trees (this study, Nouioui et al., 2018), support the conclusion that five members of this cluster, namely, *M. agarici, M. chengjingii, M. fandaimingii, M. humi*, and *M. lindanitolerans*, should be transferred to a new genus of the family *Microbacteriaceae*.

The kind and/or isomer of the diamino acids of the cell wall peptidoglycan is of taxonomically significant value in distinguishing actinomycete genera, in particular within the *Microbacteriaceae* (Evtushenko, 2012). Members of the genus *Microbacterium* contain L-lysine, D-ornithine, or diaminobutyric acid as the diagnostic diamino acid in their cell walls, depending on the strain (Suzuki and Hamada, 2012; Fidalgo et al., 2016). Only one diamino acid is found in most of the phylogenomic clusters defined in this study, but two diamino acids are present in the cell walls in some of the clusters (Suzuki and Hamada, 2012): *M. radiodurans* (D-ornithine; Zhang et al., 2010) and the other two species (L-lysine) of cluster III (*M. imperiale* clade); *M. enclense* (L-lysine; Mawlankar et al., 2015) and the other three species (D-ornithine) of cluster IV (*M. testaceum* clade); and *M. gubbeenense* (L-lysine) and the other eight species (D-ornithine) of cluster VIII (*M. gubbeenense* clade). The study strains contain D-ornithine in their cell walls, which is consistent with the type strains of cluster V (*M. liquefaciens* clade). Menaquinones with completely unsaturated (non-hydrogenated) side chains are found in members of the family *Microbacteriaceae* (Evtushenko, 2012). Members of the genus *Microbacterium* are known to contain completely unsaturated menaquinones with 10 to 14 isoprene units (Suzuki and Hamada, 2012). The study strains were shown to have MK-10 or MK-11 and MK-12 depending on the strain, similar to the seven type strains of cluster V (*M. liquefaciens* clade), except for the presence of MK-10. Other chemotaxonomic properties of the study strains, such as cell wall acyl type, polar lipid profiles, and major fatty acids, were typical for members of the genus *Microbacterium* (Suzuki and Hamada, 2012).

For the physiological and biochemical features tested in this study, the strains of groups A, C, and D showed intra-group variability of 4.4%, 2.2%, and 1.1%, respectively. In addition, some of the reference type strains gave results (1.1–12.1%) conflicting with the previously reported data, with the largest difference in *M. paraoxydans*. These results reflect that the use of the phenotypic traits should be applied at the strain level (Riesco et al., 2018) because of limited value in differentiating species (Amaral et al., 2014; Sutcliffe, 2015).

## Concluding remarks

This study was designed to provide a more stable and reliable framework for the classification of the genus *Microbacterium* through phylogenetic analyses based on the 16S rRNA gene and genome sequences. For these, the 16S rRNA gene sequences of all *Microbacterium* type strains were compared with one another and further with the corresponding genome-derived ones if available, and the phylogenetic tree was constructed with authentic and nearly complete 16S rRNA gene sequences.

Second, the overall phylogenomic clustering of the genus *Microbacterium* was performed by core genome analysis for providing a working guideline for description of new taxa in future. Third, OGRI calculations, such as OrthoANIu and dDDH, were applied to the 10 study strains and *Microbacterium* type strains for the delineation of species. Moreover, the AAI values as genus boundary were compared between the *Microbacterium* type strains. In addition, the phenotypic markers such as chemotaxonomic, physiological, and biochemical traits were examined for characterization of the study strains and closely related type strains. The results of this study support the conclusion that the 10 study strains constitute members of four new *Microbacterium* species and that several *Microbacterium* species names are later heterotypic synonyms of earlier species names. In addition, five *Microbacterium* species from the “*Microbacterium agarici* clade” should be assigned to a new genus of the family *Microbacteriaceae*.

### Taxonomic consequences: new taxa

#### Description of *Paramicrobacterium* gen. nov.

*Paramicrobacterium* **(**Gr. pref. *para-*, beside; N.L. neut. n. *Microbacterium*, a genus name; N.L. neut. n. *Paramicrobacterium* resembling the genus *Microbacterium*).

Cells are Gram-stain-positive, oxidase-positive and catalase-positive, and rod-shaped. The peptidoglycan is of type B2 with D-ornithine as diamino acid. The major menaquinone is MK-11. The presence of MK-10 or MK-12 is variable depending on species. The predominant polar lipids are diphosphatidylglycerol, phosphatidylglycerol, and an unidentified glycolipid. The major fatty acids are *anteiso*-C_15:0_, *anteiso*-C_17:0_, and *iso*-C_16:0_. The presence of *iso*-C_15:0_ as the main component is variable depending on species. The genomic DNA G+C contents are 62.0–67.3%. The type species is *Paramicrobacterium agarici*. The genus belongs to the family *Microbacteriaceae*.

#### Description of *Paramicrobacterium agarici* comb. nov.

*Paramicrobacterium agarici* (N.L. gen. masc. n. *agarici*, of *Agaricus*, the generic name of the mushroom *Agaricus blazei* from where the type strain was isolated).

Basonym: *Microbacterium agarici* Young et al. 2010

The description is as given by Young et al. (2010) and Nouioui et al. (2018). The type strain is CC-SBCK-209^T^ (= DSM 21798^T^ = CCM 7686^T^). “*Microbacterium lindanitolerans*” MNA2 (= DSM 22422 = CCM 7585) is a different strain of this species.

#### Description of *Paramicrobacterium humi* comb. nov.

*Paramicrobacterium humi* (hu'mi. L. gen. n. *humi* of earth, soil, of soil, the source of the type strain).

Basonym: *Microbacterium hum*i Young et al. 2010

The description is as given by Young et al. (2010) and Nouioui et al. (2018). The type strain is CC-12309^T^ (= DSM 21799^T^ = CCM 7687^T^).

#### Description of *Paramicrobacterium chengjingii* comb. nov.

*Paramicrobacterium chengjingii* (cheng.jing'i.i. N.L. gen. masc. n. *chengjingii*, of Professor Jing Cheng, a famous medical biophysics expert, for his important achievements and innovations in the research of biochips).

Basonym: *Microbacterium chengjingii* Zhou et al. 2021

The description is as given by Zhou et al. (2021) with following changes. The genome size of the type strain is approximately 3.62 Mbp, and its genomic G+C content is 62.0%. The type strain is HY60^T^ (= CGMCC 1.17468^T^ = GDMCC 1.1951^T^ = KACC 22102^T^).

#### Description of *Paramicrobacterium fandaimingii* comb. nov.

*Paramicrobacterium fandaimingii (*fan.dai.ming'i.i. N.L. gen. masc. n. *fandaimingii*, of Professor Daiming Fan, a well-known gastroenterologist, for his excellent research in clinical and basic research of digestive diseases and integrated medical theory).

Basonym: *Microbacterium fandaimingii* Zhou et al. 2021

The description is as given by Zhou et al. (2021) with following changes. The genome size of the type strain is approximately 3.62 Mbp, and its genomic G+C content is 63.3%. The type strain is HY82^T^ (= CGMCC 1.17469^T^ = GDMCC 1.1949^T^ = KACC 22101^T^).

#### Description of *Microbacterium aurugineum* sp. nov.

*Microbacterium aurugineum* (au.ru.gi'ne.um. L. neut. adj. *aurugineum* yellowish).

Cells are Gram-stain-positive, strictly aerobic, and short rods (0.6–0.9 x 1.1–1.7 μm). Non-motile. Catalase-positive and oxidase-negative. Cells grow well on MA, R2A, and TSA and moderately on NA. No diffusible pigments are produced. Colonies are yellow in color and reach 0.5–1.0 mm in diameter after incubation on MA for 3 days at 30°C. The temperature and pH for growth are 10–40°C (optimum, 30°C) and pH 5.0–9.0 (optimum, pH 7.0). Salt tolerance for growth is 0–7% (w/v) NaCl (optimum, 1%). Nitrate is not reduced to nitrite. Esculin degradation and gelatin hydrolysis are observed. Glucose fermentation and activities of arginine dihydrolase and urease are absent. Cells are positive for assimilation of *N*-acetylglucosamine L-arabinose, gluconate, D-glucose, malate, D-maltose, D-mannitol, D-mannose, and phenylacetate (weak). Activities of N-acetyl-β-glucosamidase, acid phosphatase, alkaline phosphatase, esterase (C4), esterase lipase (C8), α-fucosidase, β-galactosidase, α-glucosidase, β-glucosidase, β-glucuronidase (weak), lipase (C14), leucine arylamidase, α-mannosidase, naphthol-AS-BI-phosphohydrolase, and valine arylamidase are present. Acid is produced from D-arabinose, L-arabinose, arbutin, D-cellobiose, D-fructose, D-galactose, gentibiose, gluconate, D-glucose, glycerol, D-maltose, D-mannitol, D-mannose, salicin, sucrose, D-trehalose, D-turanose, and D-xylose. Acid production from D-melezitose, methyl-α-D-glucoside, L-rhamnose, and D-ribose is variable depending on the strains. The diamino acid in the cell-wall peptidoglycan is D-ornithine. The major menaquinone is MK-10, with small amounts of MK-11 and MK-12. The polar lipids contain diphosphatidylglycerol, phosphatidylglycerol, and an unidentified glycolipid. The major cellular fatty acids (>10% of the total) are *anteiso*-C_15:0_, *anteiso*-C_17:0_, *iso*-C_15:0_, and *iso*-C_16:0_.

The type strain KSW4-10^T^ (= KACC 22272^T^ = DSM 112583^T^) was isolated from dried seaweed in Gwakji Beach in Jeju, Republic of Korea. The genome size of the type strain is approximately 3.63 Mbp, and its genomic G+C content is 68.2%. Additional strains, KSW4-16 (= KACC 22273) and SSW1-7 (= KACC 22274), were isolated from dried seaweeds collected from Gwakji and Samyang Beaches in Jeju, Republic of Korea, respectively.

#### Description of *Microbacterium croceum* sp. nov.

*Microbacterium croceum* (cro.ce'um. L. neut. adj. *croceum*, saffron-colored, yellow, golden, referring to the pale-yellow color of colonies).

Cells are Gram-stain-positive, strictly aerobic, and short rods (0.4–0.6 x 0.7–1.4 μm). Non-motile. Catalase-positive and oxidase-negative. Cells grow well on MA, R2A, and TSA and moderately on NA. No diffusible pigments are produced. Colonies are yellow in color and reach 1.0 mm in diameter after incubation on MA for 3 days at 30°C. The temperature and pH for growth are 10–37°C (optimum, 30°C) and pH 4.0–9.0 (optimum, pH 7.0). Salt tolerance for growth is 0–6% (w/v) NaCl (optimum, 1%). Nitrate is not reduced to nitrite. Esculin degradation is observed. Glucose fermentation, gelatin hydrolysis, and activities of arginine dihydrolase and urease are absent. Cells are positive for assimilation of L-arabinose, D-glucose, gluconate, malate, D-maltose, D-mannitol, D-mannose, and phenylacetate (weak). Activities of N-acetyl-β-glucosamidase, acid phosphatase, alkaline phosphatase, α-chymotrypsin, cystine arylamidase, esterase (C4), esterase lipase (C8), α-fucosidase (weak), α-galactosidase, β-galactosidase, α-glucosidase, β-glucosidase, β-glucuronidase (weak), lipase (C14), leucine arylamidase, α-mannosidase (weak), naphthol-AS-BI-phosphohydrolase, trypsin, and valine arylamidase are present. Acid is produced from D-arabinose, L-arabinose, D-cellobiose, D-fructose, L-fucose, D-galactose, gentibiose, gluconate, D-glucose, glycerol, D-lyxose, D-maltose, D-mannitol, D-mannose, D-melezitose, methyl-α-D-glucoside, L-rhamnose, D-ribose, salicin, sucrose, D-trehalose, D-turanose, and D-xylose. The diamino acid in the cell-wall peptidoglycan is D-ornithine. The major menaquinone is MK-10, with small amount of MK-11. The polar lipids contain diphosphatidylglycerol, phosphatidylglycerol, an unidentified glycolipid, and an unidentified lipid. The major cellular fatty acids (>10% of the total) are *anteiso*-C_15:0_, *anteiso*-C_17:0_, and *iso*-C_16:0_.

The type strain SSW1-49^T^ (= KACC 22275^T^ = DSM 112581^T^) was isolated from dried seaweed in Samyang Beach in Jeju, Republic of Korea. The genome size is approximately 3.68 Mbp, and its genomic G+C content is 68.4%.

#### Description of *Microbacterium galbinum* sp. nov.

*Microbacterium galbinum* (gal.bi'num. L. neut. adj. *galbinum* greenish yellow).

Cells are Gram-stain-positive, strictly aerobic, and short rods (0.4–0.7 x 1.4–1.6 μm). Non-motile. Catalase-positive and oxidase-negative. Cells grow well on MA, R2A, and TSA and moderately on NA. No diffusible pigments are produced. Colonies are greenish yellow in color and reach 0.5–1.0 mm in diameter after incubation on MA for 3 days at 30°C. The temperature and pH for growth are 10–37°C (optimum, 30°C) and pH 4.0–9.0 (optimum, pH 7.0). Salt tolerance for growth is 0–6% (w/v) NaCl (optimum, 1% NaCl). Nitrate is not reduced to nitrite. Esculin degradation is observed. Glucose fermentation and activities of arginine dihydrolase and urease are absent. Cells are positive for assimilation of L-arabinose, gluconate, D-glucose, malate, D-maltose, D-mannitol, and D-mannose. Activities of N-acetyl-β-glucosamidase, acid phosphatase, alkaline phosphatase, cystine arylamidase (weak), esterase (C4), esterase lipase (C8), α-galactosidase, β-galactosidase, α-glucosidase, β-glucosidase, lipase (C14), leucine arylamidase, α-mannosidase, naphthol-AS-BI-phosphohydrolase, trypsin, and valine arylamidase are present. Acid is produced from D-arabinose, L-arabinose, D-cellobiose, D-fructose, L-fucose, D-galactose, gentibiose, gluconate, D-glucose, glycerol, D-lyxose, D-maltose, D-mannitol, D-mannose, D-melezitose, D-melibiose, methyl-α-D-glucoside, salicin, sucrose, D-trehalose, D-turanose, and D-xylose. Gelatin hydrolysis and acid production from N-acetylglucosamine are variable depending on the strains. The diamino acid in the cell wall peptidoglycan is D-ornithine. The major menaquinone is MK-10, with small amount of MK-11. The polar lipids contain diphosphatidylglycerol, phosphatidylglycerol, and an unidentified glycolipid. The major cellular fatty acids (>10% of the total) are *anteiso*-C_15:0_, *anteiso*-C_17:0_, and *iso*-C_16:0_.

The type strain KSW2-24^T^ (= KACC 22276^T^ = DSM 112584^T^) was isolated from dried seaweed in Gwakji Beach in Jeju, Republic of Korea. The genome size of the type strain is approximately 3.57 Mbp, and its genomic G+C content is 69.2%. Additional strains, KSW4-6 (= KACC 22277) and SSW1-36 (= KACC 22278), were isolated from dried seaweeds collected from Gwakji and Samyang Beaches in Jeju, Republic of Korea, respectively.

#### Description of *Microbacterium sufflavum* sp. nov.

*Microbacterium sufflavum* (suf.fla'vum. L. neut. adj. *sufflavum*, light yellow).

Cells are Gram-stain-positive, strictly aerobic, and short rods (0.7–0.9 x 1.3–2.0 μm). Non-motile. Catalase-positive and oxidase-negative. Cells grow well on MA, R2A, and TSA and moderately on NA. No diffusible pigments are produced. Colonies are yellow in color and reach 0.5–1.2 mm in diameter after incubation on MA for 3 days at 30°C. The temperature and pH for growth are 10–45°C (optimum, 30°C) and pH 4.0–9.0 (optimum, pH 7.0). Salt tolerance for growth is 0–7% (w/v) NaCl (optimum, 1% NaCl). Nitrate is not reduced to nitrite. Esculin degradation is observed. Glucose fermentation, gelatin hydrolysis, and activities of arginine dihydrolase and urease are absent. Cells are positive for assimilation of *N*-acetylglucosamine, gluconate, D-glucose, malate D-maltose, D-mannitol, and D-mannose. Activities of N-acetyl-β-glucosamidase, acid phosphatase, alkaline phosphatase, esterase (C4), esterase lipase (C8) (weak), α-fucosidase, α-glucosidase, β-glucosidase, lipase (C14), leucine arylamidase, α-mannosidase, naphthol-AS-BI-phosphohydrolase, and valine arylamidase are present. Acid is produced from D-arabinose, D-cellobiose, D-fructose, D-galactose, gentibiose, gluconate, D-glucose, glycerol, D-lyxose, D-maltose, D-mannitol, D-mannose, D-melezitose, methyl-α-D-glucoside, L-rhamnose, sucrose, D-trehalose, D-turanose, and D-xylitol. Acid produced from N-acetylglucosamine is variable depending on the strains. The diamino acid in the cell wall peptidoglycan is D-ornithine. The major menaquinone is MK-10. The polar lipids contain diphosphatidylglycerol, phosphatidylglycerol, and an unidentified glycolipid. The major cellular fatty acids (>10% of the total) are *anteiso*-C_15:0_, *anteiso*-C_17:0_, and *iso*-C_16:0_.

The type strain SSW1-47^T^ (= KACC 22279^T^ = DSM 112582^T^) was isolated from seaweed in Samyang Beach in Jeju, Republic of Korea. The genome size of the type strain is approximately 3.63 Mbp, and its genomic G+C content is 71.0%. An additional strain, SSW1-51 (= KACC 22280), was isolated from the same source.

## Data availability statement

The datasets presented in this study can be found in online repositories. The names of the repository/repositories and accession number(s) can be found below: https://www.ncbi.nlm.nih.gov/genbank/, MW703964-MW703972, MZ701896, MZ433296-MZ433298, CP078075-CP078078, JAWXG000000000-JAWXN000000000, JALPCF000000000-JALPCG000000000.

## Author contributions

SL: Data curation, Formal analysis, Investigation, Methodology, Writing – original draft, Writing – review & editing. HY: Data curation, Formal analysis, Investigation, Methodology, Software, Writing – review & editing. IK: Writing – review & editing, Project administration, Supervision.
