# Supplementary material for: Four new Microbacterium species isolated from seaweeds and reclassification of five Microbacterium species with a proposal of Paramicrobacterium gen. nov. under a genome-based framework of the genus Microbacterium
Source: Front Microbiol. 2023 Dec 18;14:1299950. doi: 10.3389/fmicb.2023.1299950 (PMC10757982; doi:10.3389/fmicb.2023.1299950)
Supplement: Supplementary file 1 [file Data_Sheet_1.zip › Supplementary table S3.docx]

**Table S3** | Origin of isolation for each type strain included in the clusters

| **Species** | **Origin of type strain** | **Reference** |
| --- | --- | --- |
| **Cluster I (*M. trichothecenolyticum* clade)** |  |  |
| *Microbacterium allomyrinae* | Insect larvae | Lee and Kim, 2023 |
| *Microbacterium* *arthrosphaerae*^*^ | Faeces of the pill millipede | Kämpfer et al., 2011 |
| *Microbacterium atlanticum* | Sediment of South Atlantic Ocean | Xie et al., 2021 |
| *Microbacterium* *aureliae*^*^ | Moon jellyfish | Kaur et al., 2016 |
| *Microbacterium cremeum* | Root soil of *Changyi Tamarix* forest marine ecological protection zone | Xie et al., 2021 |
| *Microbacterium* *flavescens* |  | Collins et al, 1983; Takeuchi and Hatano, 1998a |
| *Microbacterium hibisci* | Rhizosphere of *Hibiscus syriacus* L. | Yan et al., 2017 |
| *Microbacterium insulae* | Soil | Yoon et al., 2009 |
| *Microbacterium jejuense* | Soil | Kook et al., 2014 |
| *Microbacterium ketosireducens* | Soil | Takeuchi and Hatano, 1988b |
| *Microbacterium kyungheense* | Soil | Kook et al., 2014 |
| *Microbacterium terrae* | Soil | Takeuchi and Hatano, 1998a; Yokota et al., 1993b |
| *Microbacterium trichothecenolyticum* | Soil | Takeuchi and Hatano, 1998a; Yokota et al., 1993b |
| *Microbacterium ureisolvens* | River sediment | Cheng et al., 2019 |
| *Microbacterium yannicii* | Plant roots | Karojet et al., 2012 |
| **Cluster II (*Microbacterium* *lacticum* clade)** |  |  |
| *Microbacterium aurum* | Corn steep liquor | Yokota et al., 1993a |
| *Microbacterium dextranolyticum* | Soil | Yokota et al., 1993a |
| *Microbacterium lacticum* | Milk, dairy products | Orla-Jensen, 1919; Approved Lists, 1980 |
| *Microbacterium* *flavum* | Marine environment | Kageyama et al., 2008 |
| *Microbacterium* ***hominis*** | **Lung aspirates** | Takeuchi and Hatano, 1998b |
| *Microbacterium laevaniformans* | Activated sludge | **Dias and Bhat, 1962; Collins et al., 1983a** |
| *Microbacterium paulum* | Microfiltered milk | Bellassi et al., 2021 |
| **Cluster III (*Microbacterium* *imperiale* clade)** |  |  |
| *Microbacterium* *arborescens* | Unknown | Imai et al., 1985 |
| *Microbacterium* *imperiale* | Moss | Collins et al., 1983a |
| *Microbacterium radiodurans* | Soil | Zhang et al., 2010 |
| **Cluster IV (*Microbacterium* *testaceum* clade)** |  |  |
| *Microbacterium* *enclense* | Sediment | Mawlankar et al., 2015 |
| *Microbacterium hydrothermale* | Hydrothermal sediment | Zhang et al., 2014 |
| *Microbacterium* *proteolyticum* | Plant roots | Alves et al., 2015 |
| *Microbacterium* *testaceum* | Rice | Takeuchi and Hatano, 1998a |
| *Microbacterium zeae*^*^ | Maize stem | Gao et al., 2017 |
| **Cluster V (*Microbacterium liquefaciens* clade)** |  |  |
| *Microbacterium algeriense* | Oil production waters | Lenchi et al., 2020 |
| *Microbacterium foliorum* | Phyllosphere of grasses | Behrendt et al., 2001 |
| *Microbacterium hydrocarbonoxydans* | Oil storage cavern | Schippers et al., 2005 |
| *Microbacterium liquefaciens* | Dairy products | Collins et al., 1983; Takeuchi and Hatano, 1998a |
| *Microbacterium luteolum* | Soil | Yokota et al., 1993b; Takeuchi and Hatano, 1998a |
| *Microbacterium maritypicum* | Sea water and marine mud | ZoBell and Upham, 1944; Takeuchi and Hatano, 1998b |
| *Microbacterium oxydans* | Hospital materials | Chatelain and Second 1966; Schumann et al., 1999 |
| *Microbacterium paraoxydans* | Human blood | Laffineur et al., 2003 |
| *Microbacterium phyllosphaerae* | Phyllosphere of grasses | Behrendt et al., 2001 |
| *Microbacterium saperdae* | Dead larvae of an insect | Lysenko 1959; Takeuchi and Hatano, 1998a |
| **Cluster VI (*Microbacterium* *aerolatum* clade)** |  |  |
| *Microbacterium* *aerolatum* | Air | Zlamala et al., 2002 |
| *Microbacterium* *ginsengiterrae* | Soil | Kim et al., 2010 |
| *Microbacterium murale* | Indoor wall | Kämpfer et al., 2012 |
| *Microbacterium* *profundi* | Deep-sea sediment | Wu et al., 2008 |
| *Microbacterium tenebrionis* | Insect larvae | Lee and Kim, 2023 |
| *Microbacterium ihumii*^†^ | Human blood | Yacouba et al., 2022 |
| *Microbacterium* *panaciterrae*^*^ | Rhizosphere soil | Nguyen et al., 2015 |
| *Microbacterium* *shaanxiense*^*^ | Nodule surface of soybean | Peng et al., 2015 |
| *Microbacterium* *tumbae*^*^ | Stone chamber of ancient tumulus | Nishijima et al., 2017 |
| **Cluster VII (*Microbacterium* *resistens* clade)** |  |  |
| *Microbacterium azadirachtae* | Rhizoplane of neem seedlings | Madhaiyan et al., 2010 |
| *Microbacterium* *pseudoresistens* | Mushroom | Young et al., 2010 |
| *Microbacterium* *resistens* | Soil of rice field | Behrendt et al., 2001 |
| *Microbacterium xylanilyticum* | Biofilm | Kim et al., 2005 |
| **Cluster VIII (*Microbacterium* *gubbeenense* clade)** |  |  |
| *Microbacterium amylolyticum* | Soil | Anand et al., 2012 |
| *Microbacterium* *excoecariae* | Bark of *Excoecaria agallocha* | Chen et al., 2020 |
| *Microbacterium* *faecale* | Faeces of *Columba livia* | Chen et al., 2016 |
| *Microbacterium* *gubbeenense* | Cheese | Brennan et al., 2001 |
| *Microbacterium indicum* |  | Shivaji et al., 2007 |
| *Microbacterium* *karelineae* | Halophyte plant | Zhu et al., 2021 |
| *Microbacterium* *nanhaiense* | Sea sediment | Yan et al., 2015 |
| *Microbacterium* *sorbitolivorans* | Fermentation bed in pigpen | Meng et al., 2016 |
| *Microbacterium* *suaedae* | *Suaeda aralocaspica* | Zhu et al., 2019 |
| **Cluster IX (*Microbacterium agarici* clade)** |  |  |
| *Microbacterium agarici* | Base of the mushroom | Young et al., 2010 |
| *Microbacterium chengjingii* | Bat faeces | Zhou et al., 2021 |
| *Microbacterium* *fandaimingii* | Bat faeces | Zhou et al., 2021 |
| *Microbacterium humi* | Base of the mushroom | Young et al., 2010 |
| *Microbacterium* *lindanitolerans* | Hexachlorocyclohexane-contaminated soil | Lal et al., 2010 |
| **Cluster X** |  |  |
| *Microbacterium* *marinum* | Deep-sea water | Zhang et al., 2012 |
| *Microbacterium* *oleivorans* | Oil storage cavern | Schippers et al., 2005 |
| **Cluster XI** |  |  |
| *Microbacterium fluvii* | Driftwood | Kageyama et al., 2008 |
| *Microbacterium terricola* | Soil | Kageyama et al., 2007 |
| Cluster XII |  |  |
| *Microbacterium* *aquimaris* | Seawater | Kim et al., 2008 |
| *Microbacterium* *luteum* | Seawater | Xie et al., 2021 |
| Cluster XIII |  |  |
| *Microbacterium lushaniae* | Plateau pika | Tian et al., 2021 |
| *Microbacterium wangchenii* | Faeces of *Tibetan gazelles* | Dong et al., 2020 |
| Cluster XIV |  |  |
| *Microbacterium* *luticocti* | Sewage sludge compost | Vaz-Moreira et al., 2008 |
| *Microbacterium* *protaetiae* | Gut of insect larva | Heo et al., 2020 |
| Cluster XV |  |  |
| *Microbacterium endophyticum* | Salt-marsh plant | Alves et al., 2014 |
| *Microbacterium halimionae* | Salt-marsh plant | Alves et al., 2014 |
| Cluster XVI |  |  |
| *Microbacterium* *halophytorum* | Halophytes | Li et al., 2018 |
| *Microbacterium* *halotolerans* | Saline soil | Li et al., 2005 |
| Cluster XVII |  |  |
| *Microbacterium barkeri* | Domestic sewage | Takeuchi and Hatano, 1998a |
| *Microbacterium oryzae* | Rice field soil | Kumari et al., 2013 |

^*^Included in the cluster only based on 16S rRNA gene tree.

^†^ Not included in core genome-based phylogenetic tree.
