# Supplementary material for: Four new Microbacterium species isolated from seaweeds and reclassification of five Microbacterium species with a proposal of Paramicrobacterium gen. nov. under a genome-based framework of the genus Microbacterium
Source: Front Microbiol. 2023 Dec 18;14:1299950. doi: 10.3389/fmicb.2023.1299950 (PMC10757982; doi:10.3389/fmicb.2023.1299950)
Supplement: Supplementary file 1 [file Data_Sheet_1.zip › Supplementary Table S5.docx]

**Table S5 |** Physiological and biochemical properties that are positive or negative for the ten study strains and seven closely related type strains

Strains: 1, KSW4-10^T^; 2, KSW4-16; 3, SSW1-7; 4, SSW1-49^T^; 5, KSW2-24^T^; 6, KSW4-6; 7, SSW1-36; 8, SSW1-47^T^; 9, SSW1-51; 10, KSW4-4; 11, *Microbacterium algeriense* DSM 109018^T^; 12, M*icrobacterium liquefaciens* KACC 14464^T^; 13, *Microbacterium luteolum* KACC 14465^T^; 14, *Microbacterium maritypicum* KACC 14436^T^; 15, *Microbacterium oxydans* KACC 14467^T^; 16, *Microbacterium paraoxydans* KACC 14506^T^; 17, *Microbacterium saperdae* KACC 14469^T^. +, Positive; -, negative; w, weak.

| **Characteristic** | **1** | **2** | **3** | **4** | **5** | **6** | **7** | **8** | **9** | **10** | **11** | **12** | **13** | **14** | **15** | **16** | **17** |
| --- | --- | --- | --- | --- | --- | --- | --- | --- | --- | --- | --- | --- | --- | --- | --- | --- | --- |
| Group^*^ | A | A | A | B | C | C | C | D | D | E |  |  |  |  |  |  |  |
| Growth at: |  |  |  |  |  |  |  |  |  |  |  |  |  |  |  |  |  |
| 10°C | + | + | + | + | + | + | + | + | + | + | +^†^ | + | + | + | +^†^ | +^†^ | + |
| 50°C | - | - | - | - | - | - | - | - | - | - | -^†^ | - | - | - | - | - | - |
| pH 5 | + | + | + | + | + | + | + | + | + | + | +^†^ | + | + | + | + | + | + |
| Aesculin degradation^‡^ | + | + | + | + | + | + | + | + | + | w | + | w | + | + | + | + | + |
| Glucose fermentation^‡^ | - | - | - | - | - | - | - | - | - | - | -^†^ | - | - | - | - | - | - |
| Indole production^‡^ | - | - | - | - | - | - | - | - | - | - | - | - | - | - | - | - | - |
| Assimilation of: (API 20NE) |  |  |  |  |  |  |  |  |  |  |  |  |  |  |  |  |  |
| Adipate | - | - | - | - | - | - | - | - | - | - | - | - | - | - | - | - | - |
| Caprate | - | - | - | - | - | - | - | - | - | - | - | - | - | - | - | - | - |
| D-Glucose | + | + | + | + | + | + | + | + | + | + | + | + | + | + | + | w | + |
| D-Maltose | + | + | + | + | + | + | + | + | + | + | + | + | + | + | + | + | + |
| D-Mannitol | + | + | + | + | + | + | + | + | + | + | + | + | + | + | + | + | + |
| Acid production frpm: (API CH50) |  |  |  |  |  |  |  |  |  |  |  |  |  |  |  |  |  |
| Adonitol | - | - | - | - | - | - | - | - | - | - | - | - | - | - | - | - | - |
| L-Arabitol | - | - | - | - | - | - | - | - | - | - | - | - | - | - | - | - | - |
| D-Cellobiose | + | + | + | + | + | + | + | + | + | + | + | + | + | + | + | + | + |
| Dulcitol | - | - | - | - | - | - | - | - | - | - | - | - | - | - | - | - | - |
| Erythritol | - | - | - | - | - | - | - | - | - | - | - | - | - | - | - | - | - |
| D-Fructose | + | + | + | + | + | + | + | + | + | + | + | + | + | + | + | + | + |
| D-Fucose | - | - | - | - | - | - | - | - | - | - | - | - | - | - | - | - | - |
| D-Galactose | + | + | + | + | + | + | + | + | + | + | + | + | + | +^†^ | + | + | + |
| D-Glucose | + | + | + | + | + | + | + | + | + | + | + | + | + | + | + | + | + |
| Glycogen | - | - | - | - | - | - | - | - | - | - | - | - | - | - | - | - | - |
| Inositol | - | - | - | - | - | - | - | - | - | - | - | - | - | - | - | - | - |
| Inulin | - | - | - | - | - | - | - | - | - | - | - | - | - | - | - | - | - |
| 2-Ketogluconate | - | - | - | - | - | - | - | - | - | - | - | - | - | - | - | - | - |
| 5-Ketogluconate | - | - | - | - | - | - | - | - | - | - | - | - | - | - | - | - | - |
| D-Maltose | + | + | + | + | + | + | + | + | + | + | + | + | + | + | + | + | + |
| D-Mannitol | + | + | + | + | + | + | + | + | + | + | + | + | + | + | + | + | + |
| D-Mannose | + | + | + | + | + | + | + | + | + | + | + | + | + | + | + | + | + |
| Methyl-α-D-mannoside | - | - | - | - | - | - | - | - | - | - | - | - | - | - | - | - | - |
| Methyl-β-D-xyloside | - | - | - | - | - | - | - | - | - | - | - | - | - | - | - | - | - |
| D-Sorbitol | - | - | - | - | - | - | - | - | - | - | - | - | - | - | - | - | - |
| L-Sorbose | - | - | - | - | - | - | - | - | - | - | - | - | - | - | - | - | - |
| Starch | - | - | - | - | - | - | - | - | - | - | - | - | - | - | - | - | - |
| Sucrose | + | + | + | + | + | + | + | + | + | + | + | + | + | + | + | + | + |
| D-Tagatose | - | - | - | - | - | - | - | - | - | - | - | - | - | - | - | - | - |
| D-Trehalose | + | + | + | + | + | + | + | + | + | + | + | + | + | +^†^ | + | + | + |
| D-Turanose | + | + | + | + | + | + | + | + | + | + | + | w | + | + | + | + | + |
| L-Xylose | - | - | - | - | - | - | - | - | - | - | - | - | - | - | - | - | - |
| Enzyme activities: (API ZYM) |  |  |  |  |  |  |  |  |  |  |  |  |  |  |  |  |  |
| Acid phosphatase | + | + | + | + | + | + | + | + | + | + | + | + | + | + | + | + | + |
| Alkaline phosphatase | + | + | + | + | + | + | + | + | + | + | w | + | w | w | + | + | + |
| Esterase (C4) | + | + | + | + | + | + | + | + | + | w | + | + | + | + | + | + | + |
| Esterase lipase(C8) | w | + | + | + | + | + | + | w | w | w | + | w | + | + | + | +^†^ | w |
| Leucine arylamidase | + | + | + | + | + | + | + | + | + | + | + | + | + | + | + | + | + |
| Naphthol-AS-BI-phosphohydrolase | + | + | + | + | + | + | + | + | + | + | + | + | + | + | + | + | + |
| Nitrate reductase^‡^ | - | - | - | - | - | - | - | - | - | - | - | - | - | - | - | - | - |
| Urease^‡^ | - | - | - | - | - | - | - | - | - | - | - | - | - | - | - | - | - |
| Valine arylamidase | + | + | + | + | + | + | + | + | + | w | w | + | + | w | + | w^†^ | w |

^*^Defined in the 16S rRNA gene tree.

^†^Different from the previously reported results [*Microbacterium algeriense* (Lenchi et al., 2020), *Microbacterium maritypicum* (Takeuchi and Hatano, 1998a), *Microbacterium oxydans* (Schumann et al., 1999), and *Microbacterium paraoxydans* (Laffineur et al., 2003).

^‡^API 20NE.
