# Supplementary material for: Four new Microbacterium species isolated from seaweeds and reclassification of five Microbacterium species with a proposal of Paramicrobacterium gen. nov. under a genome-based framework of the genus Microbacterium
Source: Front Microbiol. 2023 Dec 18;14:1299950. doi: 10.3389/fmicb.2023.1299950 (PMC10757982; doi:10.3389/fmicb.2023.1299950)
Supplement: Supplementary file 1 [file Data_Sheet_1.zip › Supplementary Table S6.docx]

**Table S6 |** Differential phenotypic characteristics among the ten study strains, and between the study strains and seven closely related type strains

Strains: 1, KSW4-10^T^; 2, KSW4-16; 3, SSW1-7; 4, SSW1-49^T^; 5, KSW2-24^T^; 6, KSW4-6; 7, SSW1-36; 8, SSW1-47^T^; 9, SSW1-51; 10, KSW4-4; 11, *Microbacterium algeriense* DSM 109018^T^; 12, M*icrobacterium liquefaciens* KACC 14464^T^; 13, *Microbacterium luteolum* KACC 14465^T^; 14, *Microbacterium maritypicum* KACC 14436^T^; 15, *Microbacterium oxydans* KACC 14467^T^; 16, *Microbacterium paraoxydans* KACC 14506^T^; 17, *Microbacterium saperdae* KACC 14469^T^. +, Positive; -, negative; w, weak.

| **Characteristic** | **1** | **2** | **3** | **4** | **5** | **6** | **7** | **8** | **9** | **10** | **11** | **12** | **13** | **14** | **15** | **16** | **17** |
| --- | --- | --- | --- | --- | --- | --- | --- | --- | --- | --- | --- | --- | --- | --- | --- | --- | --- |
| Group^*^ | A | A | A | B | C | C | C | D | D | E |  |  |  |  |  |  |  |
| Growth at: |  |  |  |  |  |  |  |  |  |  |  |  |  |  |  |  |  |
| 40°C | + | + | + | - | - | - | - | + | + | + | + | - | - | - | +^†^ | + | + |
| 42°C | - | - | - | - | - | - | - | + | + | + | + | - | - | - | - | +^†^ | + |
| 45°C | - | - | - | - | - | - | - | + | + | - | + | - | - | - | **-** | **-** | **-** |
| pH 4 | - | - | - | + | + | + | + | + | + | + | - | + | + | - | + | + | + |
| 7% NaCl | + | + | + | - | - | - | - | + | + | + | - | - | - | - | + | + | - |
| Assimilation of |  |  |  |  |  |  |  |  |  |  |  |  |  |  |  |  |  |
| N-Acetyl-D-glucosamine | + | + | + | - | - | - | - | + | + | + | + | + | + | + | + | + | + |
| L-Arabinose | + | + | + | + | + | + | + | - | - | - | +^†^ | - | + | - | - | - | + |
| Citrate | - | - | - | - | - | - | - | - | - | + | - | w | - | w | + | w | - |
| Gluconate | + | + | + | + | + | + | + | + | + | + | + | + | - | + | + | + | + |
| Malate | + | + | + | + | + | + | + | + | + | - | - | + | + | + | + | - | + |
| D-Mannose | + | + | + | + | + | + | + | + | + | + | + | - | + | + | + | + | + |
| Phenylacetate | w | w | w | w | - | - | - | - | - | - | - | - | - | -^†^ | - | - | - |
| Acid roduction from: (API 50CH) |  |  |  |  |  |  |  |  |  |  |  |  |  |  |  |  |  |
| N-Acetyl-glucosamine | - | - | - | - | - | - | + | + | - | - | - | - | + | - | - | -^†^ | - |
| Amygdalin | - | - | - | - | - | - | - | - | - | - | - | - | + | - | - | - | - |
| Arbutin | + | + | + | - | - | - | - | - | - | - | + | + | + | + | + | - | - |
| D-Arabinose | + | + | + | + | + | + | + | + | + | + | + | + | + | + | + | + | - |
| L-Arabinose | + | + | + | + | + | + | + | - | - | - | + | - | + | - | - | - | - |
| D-Arabitol | - | - | - | - | - | - | - | - | - | - | - | - | + | - | - | - | - |
| L-Fucose | - | - | - | + | + | + | + | + | + | + | + | - | + | - | + | + | - |
| Gentibiose | + | + | + | + | + | + | + | + | + | + | + | - | + | - | + | + | + |
| Gluconate | + | + | + | + | + | + | + | - | - | - | - | - | - | - | - | -^†^ | - |
| Glycerol | + | + | + | + | + | + | + | + | + | + | - | + | + | + | + | + | - |
| D-Lactose | - | - | - | - | - | - | - | - | - | - | - | + | + | **-** | - | -^†^ | - |
| D-Lyxose | - | - | - | + | + | + | + | + | + | - | - | - | + | - | - | - | + |
| D-Melezitose | + | - | + | + | + | + | + | + | + | + | + | - | + | +^†^ | + | + | + |
| D-Melibiose | - | - | - | - | + | + | + | - | - | - | - | - | + | - | - | - | - |
| Methyl-α-D-glucoside | + | - | + | + | + | + | + | + | + | - | - | + | + | + | + | -^†^ | - |
| L-Raffinose | - | - | - | - | - | - | - | - | - | - | - | - | + | +^†^ | - | - | - |
| L-Rhamnose | + | + | - | + | - | - | - | + | + | + | + | - | + | - | + | + | - |
| D-Ribose | + | + | - | + | - | - | - | - | - | - | + | - | - | - | + | -^†^ | - |
| Salicin | + | + | + | + | + | + | + | - | - | - | + | + | + | + | + | - | - |
| D-Xylitol | - | - | - | - | - | - | - | + | + | - | - | - | - | - | + | - | - |
| D-Xylose | + | + | + | + | + | + | + | - | - | - | + | - | + | - | - | - | - |
| Enzyme activities: (API ZYM) |  |  |  |  |  |  |  |  |  |  |  |  |  |  |  |  |  |
| N-Acetyl- β-glucosamidase | + | + | + | + | + | + | + | + | + | + | + | - | + | + | + | + | + |
| Arginine dihydrolase^‡^ | - | - | - | - | - | - | - | - | - | - | - | + | - | - | - | - | + |
| α-Chymotrypsin | - | - | - | + | - | - | - | - | - | - | - | - | - | - | + | - | - |
| Cystine arylamidase | - | - | - | + | w | w | w | - | - | w | - | - | + | - | w | w^†^ | w |
| α-Fucosidase | + | + | + | w | - | - | - | + | + | + | - | - | - | + | + | + | - |
| α-Galactosidase | - | - | - | + | + | + | + | - | - | - | - | - | + | - | - | - | + |
| β-Galactosidase | + | + | + | + | + | + | + | - | - | + | w | w | + | + | w | + | + |
| Gelatinase^‡^ | + | + | w | - | + | + | - | - | - | + | - | + | + | + | + | + | - |
| α-Glucosidase | + | + | + | + | + | + | + | + | + | + | + | + | + | + | + | + | + |
| β-Glucosidase | + | + | + | + | + | + | + | + | + | + | + | + | + | + | + | +^†^ | + |
| β-Glucuronidase | w | w | w | w | - | - | - | - | - | - | - | - | - | - | - | - | - |
| Lipase (C14) | + | + | + | + | + | + | + | + | + | w | - | - | - | - | w | w | - |
| α-Mannosidase | + | + | + | w | + | + | + | + | + | + | + | + | + | + | + | + | - |
| Trypsin | - | - | - | + | + | + | + | - | - | - | - | - | - | - | + | - | - |

^*^Defined in the 16S rRNA gene tree.

^†^Different from the previously reported results [*Microbacterium algeriense* (Lenchi et al., 2020), *Microbacterium maritypicum* (Takeuchi and Hatano, 1998a), *Microbacterium oxydans* (Schumann et al., 1999), and *Microbacterium paraoxydans* (Laffineur et al., 2003).

^‡^API 20NE.
