# Supplementary material for: Four new Microbacterium species isolated from seaweeds and reclassification of five Microbacterium species with a proposal of Paramicrobacterium gen. nov. under a genome-based framework of the genus Microbacterium
Source: Front Microbiol. 2023 Dec 18;14:1299950. doi: 10.3389/fmicb.2023.1299950 (PMC10757982; doi:10.3389/fmicb.2023.1299950)
Supplement: Supplementary file 1 [file Data_Sheet_1.zip › Supplementary Table S7.docx]

**Table S7 |** Chemotaxonomic characteristics of representatives of the study strains and seven closely related type strains

D-Orn, D-Ornithine; DPG, diphosphatidylglycerol; PG, phosphatidylglycerol; GL, unidentified glycolipid; L, unidentified lipid; MK, menaquinone; *ai*, *anteiso*; *i,* *iso*; ND, not determined.

| **Strain** | **Diamino acid in cell wall** | **Polar lipids** | **Menaquinones** | | **Major fatty acids**  **(>10% of the total)^*^** | **Reference(s)** | |
| --- | --- | --- | --- | --- | --- | --- | --- |
| KSW4-10^T^ | D-Orn | DPG, PG, GL, | | MK-10, MK-11, MK-12 | *ai*-C_15:0_, *ai*-C_17:0_, *i*-C_15:0_, *i*-C_16:0_ | | This study |
| SSW1-49^T^ | D-Orn | DPG, PG, GL, L | | MK-10, MK-11 | *ai*-C_15:0_, *ai*-C_17:0_, *i*-C_16:0_ | | This study |
| KSW2-24^T^ | D-Orn | DPG, PG, GL | | MK-10, MK-11 | *ai*-C_15:0_, *ai*-C_17:0_, *i*-C_16:0_ | | This study |
| SSW1-47^T^ | D-Orn | DPG, PG, GL, | | MK-10 | *ai*-C_15:0_, *ai*-C_17:0_, *i*-C_16:0_ | | This study |
| *Microbacterium algeriense* DSM 109018^T^ | ND | DPG, PG, GL, 6L | | MK-11, MK-12 | *ai*-C_15:0_, *ai*-C_17:0_, *i*-C_16:0_ | | Lenchi et al. (2020) |
| M*icrobacterium liquefaciens* KACC 14464^T^ | D-Orn | DPG, PG, GL | | MK-11, MK-12 | *ai*-C_15:0_, *ai*-C_17:0_ | | Collins et al. (1983), Takeuchi and Hatano (1998b) |
| *Microbacterium luteolum* KACC 14465^T^ | D-Orn | DPG, PG, GL | | MK-12 | *ai*-C_15:0_, *i*-C_16:0_, *ai*-C_17:0_, *iso*-C_15:0_, *anteiso*-C_15:1_ A | | Yokota et al. (1993b), Takeuchi and Hatano (1998b) |
| *Microbacterium maritypicum* KACC 14436^T^ | D-Orn | ND | | MK-11, MK-12 | *ai*-C_15:0_, *i*-C_16:0_ | | Takeuchi and Hatano (1998a) |
| *Microbacterium oxydans* KACC 14467^T^ | D-Orn | DPG, PG, GL | | MK-11, MK-12 | *ai*-C_15:0_, *i*-C_16:0_, *ai*-C_17:0_ | | Schumann et al. (1999) |
| *Microbacterium paraoxydans* KACC 14506^T^ | ND | ND | | ND | *ai*-C_15:0_, *ai*-C_17:0_, *i*-C_16:0_ | | Laffineur et al. (2003) |
| *Microbacterium saperdae* KACC 14469^T^ | D-Orn | DPG, PG, GL | | MK-11, MK-12 | *ai*-C_15:0_, *ai*-C_17:0_, *i*-C_16:0_ | | Collins et al. (1983), Takeuchi and Hatano (1998b) |

^*^Determined in this study. The major fatty acids were arranged in the decreasing order of relative proportions.
