# Supplementary material for: Four new Microbacterium species isolated from seaweeds and reclassification of five Microbacterium species with a proposal of Paramicrobacterium gen. nov. under a genome-based framework of the genus Microbacterium
Source: Front Microbiol. 2023 Dec 18;14:1299950. doi: 10.3389/fmicb.2023.1299950 (PMC10757982; doi:10.3389/fmicb.2023.1299950)
Supplement: Supplementary file 1 [file Data_Sheet_1.zip › Supplementary Table S8.docx]

**Table S8 |** Cellular fatty acid profiles of the ten study strains and seven closely related type strains

Strains: 1, KSW4-10^T^; 2, KSW4-16; 3, SSW1-7; 4, SSW1-49^T^; 5, KSW2-24^T^; 6, KSW4-6; 7, SSW1-36; 8, SSW1-47^T^; 9, SSW1-51; 10, KSW4-4; 11, *Microbacterium algeriense* DSM 109018^T^; 12, M*icrobacterium liquefaciens* KACC 14464^T^; 13, *Microbacterium luteolum* KACC 14465^T^; 14, *Microbacterium maritypicum* KACC 14436^T^; 15, *Microbacterium oxydans* KACC 14467^T^; 16, *Microbacterium paraoxydans* KACC 14506^T^; 17, *Microbacterium saperdae* KACC 14469^T^. Bold types represent major fatty acids (>10% of the total).

| **Fatty acid** | **1** | **2** | **3** | **4** | **5** | **6** | **7** | **8** | **9** | **10** | **11** | **12** | **13** | **14** | **15** | **16** | **17** |
| --- | --- | --- | --- | --- | --- | --- | --- | --- | --- | --- | --- | --- | --- | --- | --- | --- | --- |
| Group^*^ | A | A | A | B | C | C | C | D | D | E |  |  |  |  |  |  |  |
| Saturated |  |  |  |  |  |  |  |  |  |  |  |  |  |  |  |  |  |
| C_16:0_ | 1.2 | 1.0 | 1.0 | 1.1 | 0.9 | 1.4 | 1.1 | 0.9 | 1.4 | 0.8 | 1.3 | 0.7 | 0.7 | 1.0 | 1.8 | 0.9 | 1.0 |
| *Iso*-branched |  |  |  |  |  |  |  |  |  |  |  |  |  |  |  |  |  |
| *Iso*-C_14:0_ | - | - | - | 0.5 | - | 0.4 | 0.9 | 0.4 | 0.6 | - | 0.5 | 0.2 | 1.1 | 0.8 | 0.5 | 0.8 | - |
| *Iso*-C_15:1_ G | - | - | - | - | - | - | 0.7 | 0.5 | 0.7 | - | - | - | - | - | - | - | - |
| *Iso*-C_15:0_ | **13.7** | **13.6** | **15.8** | 5.0 | 9.9 | 7.7 | 9.7 | 7.4 | 8.8 | 6.2 | 4.8 | 4.4 | **12.1** | 5.8 | 5.5 | 5.4 | 3.1 |
| *Iso*-C_16:0_ | **11.9** | **11.2** | **10.6** | **17.7** | **16.0** | **19.6** | **25.4** | **16.3** | **15.4** | **14.1** | **14.2** | 9.4 | **24.5** | **14.3** | **23.3** | **15.8** | **19.3** |
| *Iso*-C_17:0_ | 4.6 | 4.6 | 5.1 | 2.2 | 3.3 | 5.0 | 3.9 | 2.6 | 2.7 | 2.3 | 1.8 | 2.2 | 2.7 | 0.3 | 2.2 | 1.2 | 2.2 |
| *Anteiso*-branched |  |  |  |  |  |  |  |  |  |  |  |  |  |  |  |  |  |
| *Anteiso*-C_15:1_ A | 4.6 | 5.4 | 5.6 | 9.4 | 2.0 | - | 6.6 | 6.6 | 8.1 | 4.3 | 1.3 | 8.0 | **10.6** | 1.1 | 4.7 | 5.6 | 8.1 |
| *Anteiso*-C_15:0_ | **41.7** | **41.9** | **41.0** | **42.4** | **45.1** | **40.0** | **36.7** | **40.0** | **41.3** | **43.6** | **51.7** | **41.5** | **36.6** | **68.1** | **42.3** | **46.0** | **40.3** |
| *Anteiso*-C_17:0_ | **22.4** | **22.3** | **20.9** | **21.8** | **22.9** | **26.2** | **14.9** | **24.8** | **21.0** | **28.7** | **24.6** | **33.7** | **13.7** | 8.1 | **19.7** | **24.4** | **26.1** |

^*^Defined in the 16S rRNA gene tree.
